# Cost-effectiveness of a Novel Lipoarabinomannan Test for Tuberculosis in Patients With Human Immunodeficiency Virus

**DOI:** 10.1093/cid/ciaa1698

**Published:** 2020-11-17

**Authors:** Krishna P Reddy, Claudia M Denkinger, Tobias Broger, Nicole C McCann, Ankur Gupta-Wright, Andrew D Kerkhoff, Pamela P Pei, Fatma M Shebl, Katherine L Fielding, Mark P Nicol, C Robert Horsburgh, Graeme Meintjes, Kenneth A Freedberg, Robin Wood, Rochelle P Walensky

**Affiliations:** 1 Medical Practice Evaluation Center, Massachusetts General Hospital, Boston, Massachusetts, USA; 2 Division of Pulmonary and Critical Care Medicine, Massachusetts General Hospital, Boston, Massachusetts, USA; 3 Harvard Medical School, Boston, Massachusetts, USA; 4 Foundation for Innovative New Diagnostics, Geneva, Switzerland; 5 Division of Infection and Immunity, University College London, London, United Kingdom; 6 Clinical Research Department, London School of Hygiene and Tropical Medicine, London, United Kingdom; 7 Malawi-Liverpool Wellcome Trust Clinical Research Program, Blantyre, Malawi; 8 Division of HIV, Infectious Diseases, and Global Medicine, Zuckerberg San Francisco General Hospital and Trauma Center, University of California, San Francisco, San Francisco, California, USA; 9 TB Centre, London School of Hygiene and Tropical Medicine, London, United Kingdom; 10 School of Public Health, University of the Witwatersrand, Johannesburg, South Africa; 11 Infection and Immunity, University of Western Australia, Perth, Australia; 12 Department of Epidemiology, Boston University School of Public Health, Boston, Massachusetts, USA; 13 Section of Infectious Diseases, Department of Medicine, Boston University School of Medicine, Boston, Massachusetts, USA; 14 Department of Medicine, University of Cape Town, Cape Town, South Africa; 15 Wellcome Center for Infectious Diseases Research in Africa and Institute of Infectious Disease and Molecular Medicine, University of Cape Town, Cape Town, South Africa; 16 Division of General Internal Medicine, Massachusetts General Hospital, Boston, Massachusetts, USA; 17 Division of Infectious Diseases, Massachusetts General Hospital, Boston, Massachusetts General Hospital, Massachusetts, USA; 18 Department of Health Policy and Management, Harvard T. H. Chan School of Public Health, Boston, Massachusetts, USA; 19 Desmond Tutu HIV Foundation, University of Cape Town, Cape Town, South Africa

**Keywords:** tuberculosis, HIV, diagnosis, lipoarabinomannan, cost-effectiveness

## Abstract

**Background:**

A novel urine lipoarabinomannan assay (FujiLAM) has higher sensitivity and higher cost than the first-generation AlereLAM assay. We evaluated the cost-effectiveness of FujiLAM for tuberculosis testing among hospitalized people with human immunodeficiency virus (HIV), irrespective of symptoms.

**Methods:**

We used a microsimulation model to project clinical and economic outcomes of 3 testing strategies: (1) sputum Xpert MTB/RIF (*Xpert*), (2) sputum Xpert plus urine AlereLAM (*Xpert+AlereLAM*), (3) sputum Xpert plus urine FujiLAM (*Xpert+FujiLAM*). The modeled cohort matched that of a 2-country clinical trial. We applied diagnostic yields from a retrospective study (yields for *Xpert*/*Xpert+AlereLAM*/*Xpert+FujiLAM* among those with CD4 <200 cells/µL: 33%/62%/70%; among those with CD4 ≥200 cells/µL: 33%/35%/47%). Costs of Xpert/AlereLAM/FujiLAM were US$15/3/6 (South Africa) and $25/3/6 (Malawi). *Xpert+FujiLAM* was considered cost-effective if its incremental cost-effectiveness ratio (US$/year-of-life saved) was <$940 (South Africa) and <$750 (Malawi). We varied key parameters in sensitivity analysis and performed a budget impact analysis of implementing FujiLAM countrywide.

**Results:**

Compared with *Xpert+AlereLAM*, *Xpert+FujiLAM* increased life expectancy by 0.2 years for those tested in South Africa and Malawi. *Xpert+FujiLAM* was cost-effective in both countries. *Xpert+FujiLAM* for all patients remained cost-effective compared with sequential testing and CD4-stratified testing strategies. FujiLAM use added 3.5% (South Africa) and 4.7% (Malawi) to 5-year healthcare costs of tested patients, primarily reflecting ongoing HIV treatment costs among survivors.

**Conclusions:**

FujiLAM with Xpert for tuberculosis testing in hospitalized people with HIV is likely to increase life expectancy and be cost-effective at the currently anticipated price in South Africa and Malawi. Additional studies should evaluate FujiLAM in clinical practice settings.

## Teaser

Testing hospitalized patients with human immunodeficiency virus (HIV) in South Africa and Malawi for tuberculosis by the novel FujiLAM urine assay is likely to increase life expectancy and be cost-effective. These results can inform decisions about implementing FujiLAM testing.

Tuberculosis (TB) is the leading cause of death of people with human immunodeficiency virus (PWH) worldwide [1]. In sub-Saharan Africa, TB accounts for approximately 40% of hospital deaths among PWH, and half of these are undiagnosed before death [2, 3]. Sputum-based diagnostics, the current standard, suffer from the inability of some patients to produce sputum, the low sensitivity of smear, and the cost of molecular diagnostics. Moreover, extrapulmonary TB may be missed by sputum-based testing.

Urine-based assays for lipoarabinomannan (LAM) are a promising TB testing approach. Testing hospitalized PWH using a first-generation LAM lateral flow assay (Determine TB-LAM; Alere [hereafter called AlereLAM]) increases TB diagnostic yield and, in some subgroups, reduces mortality [4, 5]. However, AlereLAM’s limited sensitivity hinders more widespread clinical benefit.

The next-generation Fujifilm SILVAMP TB-LAM assay (FujiLAM) offers higher sensitivity than AlereLAM for TB detection [6–8]. A study using biobanked urine from hospitalized PWH in South Africa found FujiLAM’s sensitivity against a microbiologic reference standard to be 70%, compared with 42% for AlereLAM [6]. Specificity for both assays was over 90%. Although both can provide a result in under 1 hour without additional instrumentation, FujiLAM involves additional steps, time, and cost compared with AlereLAM [6].

Weighing additional TB detections and potential prevented deaths against additional costs is critical in deciding whether to implement FujiLAM in resource-limited settings. We therefore performed a cost-effectiveness analysis of urine FujiLAM added to sputum Xpert for TB testing among hospitalized PWH in South Africa and Malawi.

## METHODS

### Analytic Overview

We used the Cost-Effectiveness of Preventing AIDS Complications (CEPAC) International model, a validated microsimulation of human immunodeficiency virus– (HIV-) and TB-related disease and treatment [9–11]. The population of interest was adults with HIV, regardless of CD4 count or symptoms, hospitalized in medical units. We compared 3 TB testing strategies: (1) sputum Xpert alone (*Xpert*), (2) sputum Xpert and urine AlereLAM (*Xpert+AlereLAM*), and (3) sputum Xpert and urine FujiLAM (*Xpert+FujiLAM*). To attain stable per-person results, we modeled cohorts of 1 million hospitalized PWH separately in South Africa and Malawi. We based our modeled population on participants in the Rapid Urine-based Screening for Tuberculosis to Reduce AIDS-related Mortality in Hospitalized Patients in Africa (STAMP) trial in South Africa and Malawi, in which hospitalized PWH, irrespective of CD4 count or symptoms, were tested for TB by either sputum Xpert or sputum Xpert plus urine Xpert plus urine AlereLAM [5, 12]. While the STAMP trial represented our target population, it did not include FujiLAM. Therefore, we based performance characteristics of all diagnostic assays on a study that used biobanked specimens from hospitalized PWH in South Africa [6].

Model outcomes included mortality, life expectancy, and TB- and HIV-related costs from the health system perspective. The primary outcome was the incremental cost-effectiveness ratio (ICER)—the difference in lifetime healthcare costs (2017 US dollars [USD]) divided by the difference in life expectancy—between testing strategies. A strategy was strongly dominated if it resulted in lower life expectancy than a less costly strategy. A strategy was weakly dominated if it resulted in a higher ICER than a strategy that provided higher life expectancy [13]. Because second-line antiretroviral therapy (ART) is relatively expensive but implemented and recommended in national HIV care guidelines in both South Africa and Malawi, we defined an ICER less than that of second-line ART as cost-effective (ie, offering good value) (Supplementary Text) [14–16]. These thresholds were USD 940 per year-of-life saved (YLS) in South Africa and USD 750 per YLS in Malawi [9].

### Model Overview

In this analysis, simulated PWH enter the model upon TB testing and are tracked monthly until death. Initially, the model draws randomly from user-defined characteristics in each country, such as distributions of CD4 count and TB status [10]. The model tracks clinical outcomes and costs as each individual transition through various “states” of TB and HIV disease and treatment. Details about the model, validation, and treatment parameters are provided in the Supplementary Text, Supplementary Figure 1, Supplementary Table 1, and at massgeneral.org/medicine/mpec/research/cpac-model.

#### Tuberculosis Diagnostics

In the model, TB can be diagnosed from a positive test result. After diagnosis, individuals start treatment for drug-susceptible or multidrug-resistant TB (Supplementary Text). In case of only negative microbiological tests, empiric treatment can be initiated according to patterns in local practice or in studies.

### Input Parameters

#### Tuberculosis Diagnostics

We characterized the simulated population using STAMP trial data [5] (Table 1). Diagnostic data came from a cohort of hospitalized PWH, all of whom were tested for TB regardless of CD4 count or symptoms [6, 17]. We used CD4-stratified (<200 cells/μL or ≥200 cells/μL) diagnostic performance data that were not included in the published report (Table 1).

**Table 1.** Model Input Parameters

	South Africa	Malawi	Deterministic Sensitivity Analysis Range	References
Cohort characteristics				
Age, median [IQR], years	37 [30–46]	38 [32–47]	…	[5]
Men/women, %	50/50	37/63	…	[5]
CD4 count at admission, median [IQR], cells/µL	236 [70–445]	219 [86–431]	…	[5]
TB prevalence,^a^ %	29	24	15–45^b^	[5, 9, 27, 28]
MDR-TB prevalence among those with TB, %	3	1	1–7 (South Africa); 0.5–5 (Malawi)	[5, 29]
Patients able to provide sputum, %	50	50	30–90^b^	Assumption [5, 6, 25]
Probability of empiric treatment, *Xpert*,^c^ %	11	11	0–20^b^	[5, 30]
Probability of empiric treatment, *Xpert+AlereLAM* and *Xpert+FujiLAM*, %	10	10	0–20^b^	[5, 6]
Loss to follow-up from TB care after hospital discharge, %/month	3.6	3.6	50–200% of base case value^b^	[31, 32]
Mortality				
Death from untreated TB, monthly probability	0.086	0.086	25–200% of base case value^b^	[33, 34]
Death from AIDS (besides TB), CD4-dependent, monthly probability	6.2 × 10–5–0.2	6.2 × 10–5–0.2	…	[35, 36]
Cost of treatment^d^				
DS-TB treatment cost, monthly (6-month duration), USD	$7	$7	…	[37]
MDR-TB treatment cost, monthly (24-month duration), USD	$231	$231	…	[37]
First-line ART costs (TDF/3TC/EFV), monthly, USD	$11	$11	50–75% of base case value	[38]
Cost of TB diagnostic assay, per-test (USD)				
Sputum Xpert^e^	$15	$25	…	[19, 20]
Urine AlereLAM	$3	$3	…	[21]
Urine FujiLAM	$6	$6	$3–20	Estimate
	**Sensitivity**	**Specificity**		
Performance characteristics of diagnostic assays and strategies				
Diagnostic assay^f^				
Sputum Xpert, CD4 <200/≥200 cells/µL	65%/65%	98%/98%	…	[6], Assumption
Urine AlereLAM CD4 <200/≥200 cells/µL	48%/2%	97%/99%	…	[6], Assumption
Urine FujiLAM, CD4 <200/≥200 cells/µL	62%/23%	94%/98%	Sensitivity: 48%/8% to 77%/38%; specificity: 75–90%	[6], Assumption
Xpert Ultra, CD4 <200/≥200 cells/µL	77%/77%	96%/96%	…	[23]
	**Diagnostic Yield**			
Diagnostic strategy^f^				
*Xpert*, CD4 <200/≥200 cells/µL	33%/33%		…	[6], Assumption
*Xpert+AlereLAM*, CD4 <200/≥200 cells/µL	62%/35%		−20% to +20% of base case value	[6], Assumption
*Xpert+FujiLAM*, CD4 <200/≥200 cells/µL	70%/47%		−20% to +20% of base case value	[6], Assumption

Abbreviations: ART, antiretroviral therapy; DS, drug-susceptible; EFV, efavirenz; HIV, human immunodeficiency virus; IQR, interquartile range; LAM, lipoarabinomannan; MDR, multidrug-resistant; TB, tuberculosis; TDF, tenofovir; USD, 2017 US dollars; 3TC, lamivudine.

^a^TB prevalence is the true prevalence among the simulated group of hospitalized patients with HIV.

^b^These parameters were also examined in probabilistic sensitivity analysis using beta distributions (Supplementary Text).

^c^Those who were diagnosed clinically without microbiologic confirmation were empirically treated in the first month of model simulation.

^d^We assumed that costs of TB drugs and ART drugs were equal across countries because they are imported across countries. Costs shown here are for drugs only.

^e^Xpert cost in a Malawi-specific costing study was higher than the cost reported in South African studies and by the South Africa National Health Laboratory Service [19]. This is due to factors such as different costs of maintenance and repair and different economies of scale.

^f^The indicated sensitivity of each assay is the sensitivity among those who provided a specimen and is independent of other test results. Italics reflect a diagnostic strategy rather than a single test. The diagnostic strategy yields applied in the model accounted for nonprovision of sputum specimens and for concordance between test results—eg, adding FujiLAM would increase diagnostic yield only if FujiLAM detected additional TB cases not detected by Xpert. In multitest strategies, we applied the lowest specificity of any individual test.

For performance characteristics of each testing strategy (*Xpert*, *Xpert+AlereLAM*, and *Xpert+FujiLAM*) in the model, we applied diagnostic yields, calculated as follows: the number of subjects who had a correct positive TB result by at least 1 test in the strategy, divided by the number of subjects diagnosed with TB by the composite reference standard, all from Broger et al [6] (Supplementary Text). The diagnostic yield accounted for the number of subjects able to provide a specimen and the incremental diagnostic yield of the LAM tests over sputum Xpert (ie, the additional cases detected by LAM that were not diagnosed by sputum Xpert) (Supplementary Text). We assumed 50% of individuals would provide a sputum specimen (Supplementary Text). The diagnostic yields applied in our base case analysis were as follows for CD4 <200/≥200 cells/µL: *Xpert*, 33%/33%; *Xpert+AlereLAM*, 62%/35%; *Xpert+FujiLAM*, 70%/47% (Table 1 and Supplementary Text). We applied specificity of Xpert from a meta-analysis and Horne et al [18] specificity of AlereLAM and FujiLAM from Broger et al [6].

#### Costs

In South Africa/Malawi, sputum Xpert costs were USD 15/25 and urine AlereLAM costs were USD 3/3 [9, 19–21]. Although the price of urine FujiLAM has not yet been established, we used in the base case a best estimate of the anticipated cost, USD 6, which is also in line with the World Health Organization’s (WHO’s) target product profile [22]. We varied this cost from USD 3 to USD 20 in sensitivity analysis. We included additional TB and HIV care costs (Supplementary Text).

### Uncertainty Analysis

#### Deterministic Sensitivity Analysis

We performed 1-way and multi-way deterministic sensitivity analysis by varying key parameters across ranges (Table 1). When varying FujiLAM sensitivity, we accounted for its impact on the diagnostic yield of *Xpert+FujiLAM* (Supplementary Text).

#### Probabilistic Sensitivity Analysis

In a probabilistic sensitivity analysis, we simultaneously varied several parameters across beta distributions to understand how results would vary in other settings or scenarios (Supplementary Text). These parameters were TB prevalence, sputum provision, empiric TB treatment, loss to follow-up from TB care, and death from untreated TB. We used the results to generate a cost-effectiveness acceptability curve.

### Alternative Testing Strategies

We evaluated alternative TB testing strategies, including the following: solo strategies (Xpert, AlereLAM, or FujiLAM alone), sequential strategies (whereby a urine LAM test is done and, if positive, is followed by sputum Xpert for rifampicin-resistance testing), and CD4-stratified strategies (sputum Xpert plus urine LAM for those with CD4 <200 cells/μL; sputum Xpert alone for those with CD4 ≥200 cells/μL). We compared the outcomes of these strategies with those of the 3 strategies of the base case, generating a cost-effectiveness frontier; strategies that lie on the frontier are economically efficient. We also evaluated a scenario in which Xpert Ultra was substituted for Xpert in each of the 3 base case strategies, offering higher sensitivity and lower specificity at similar cost to Xpert (Table 1 and Supplementary Text) [23].

### Budget Impact Analysis

We conducted a budget impact analysis of performing *Xpert+FujiLAM* instead of *Xpert* countrywide among all hospitalized PWH over 1 year and 5 years, assuming 500 000 and 70 000 annual hospitalizations among PWH in South Africa and Malawi (Supplementary Text) [9]. We considered FujiLAM per-test cost of either USD 6 or USD 20.

## RESULTS

### Base Case

Lipoarabinomannan testing strategies increased the number of positive TB results (Supplementary Table 2). In the base case analysis in South Africa and Malawi, *Xpert+AlereLAM* and *Xpert+FujiLAM* both reduced 2-year mortality and increased life expectancy compared with *Xpert* (Table 2). Undiscounted life expectancy with *Xpert*/*Xpert+AlereLAM*/*Xpert+FujiLAM* was 13.2/13.7/13.9 years in South Africa and 12.7/13.1/13.3 years in Malawi. With regard to cost-effectiveness, *Xpert+AlereLAM* was weakly dominated by the more effective *Xpert+FujiLAM*. In both countries, *Xpert+FujiLAM* was cost-effective compared with *Xpert*, with an ICER of USD 830/YLS in South Africa and USD 440/YLS in Malawi (Table 2).

**Table 2.** Base Case Model Clinical, Cost, and Cost-Effectiveness Results

Testing Strategy	Mortality at 2 Years, %	Life-years, Discounted^a^ (Undiscounted)	Cost, USD, Discounted^a,b^	ICER, USD/YLS^c^
South Africa				
*Xpert*	35.8	8.9 (13.2)	8230	…
*Xpert+AlereLAM*	33.3	9.2 (13.7)	8500	Dominated^d^
*Xpert+FujiLAM*	32.1	9.4 (13.9)	8640	830
Malawi				
*Xpert*	38.9	8.5 (12.7)	3540	…
*Xpert+AlereLAM*	37.2	8.8 (13.1)	3640	Dominated^d^
*Xpert+FujiLAM*	36.2	8.9 (13.3)	3710	440

Abbreviations: ICER, incremental cost-effectiveness ratio; LAM, lipoarabinomannan; USD, 2017 US dollars; YLS, year-of-life saved.

^a^Discounted 3% per year [39].

^b^This reflects lifetime healthcare costs.

^c^The ICER is the difference between 2 strategies in discounted costs divided by the difference in discounted life-years. The displayed life-years and costs are rounded, but the ICER was calculated using nonrounded life-years and costs. We considered a strategy cost-effective if its ICER was less than USD 940/YLS in South Africa and less than USD 750/YLS in Malawi (the ICERs of second-line antiretroviral therapy in these countries).

^d^This indicates “weak dominance” [40]. The ICER of *Xpert+AlereLAM* versus *Xpert* was higher (less attractive) than the ICER of *Xpert+FujiLAM* versus *Xpert+AlereLAM*, indicating an inefficient use of resources.

### Sensitivity and Uncertainty Analysis

When varying key parameters in 1-way sensitivity analysis, *Xpert+AlereLAM* was weakly dominated by *Xpert+FujiLAM* in most analyses in South Africa and Malawi (Supplementary Table 3). *Xpert+FujiLAM* remained cost-effective compared with *Xpert* in all these analyses, except in South Africa when the *Xpert+FujiLAM* yield was decreased by 20 percentage points (ie, <50%/<27% for low/high CD4 count).

**Figure 1.**
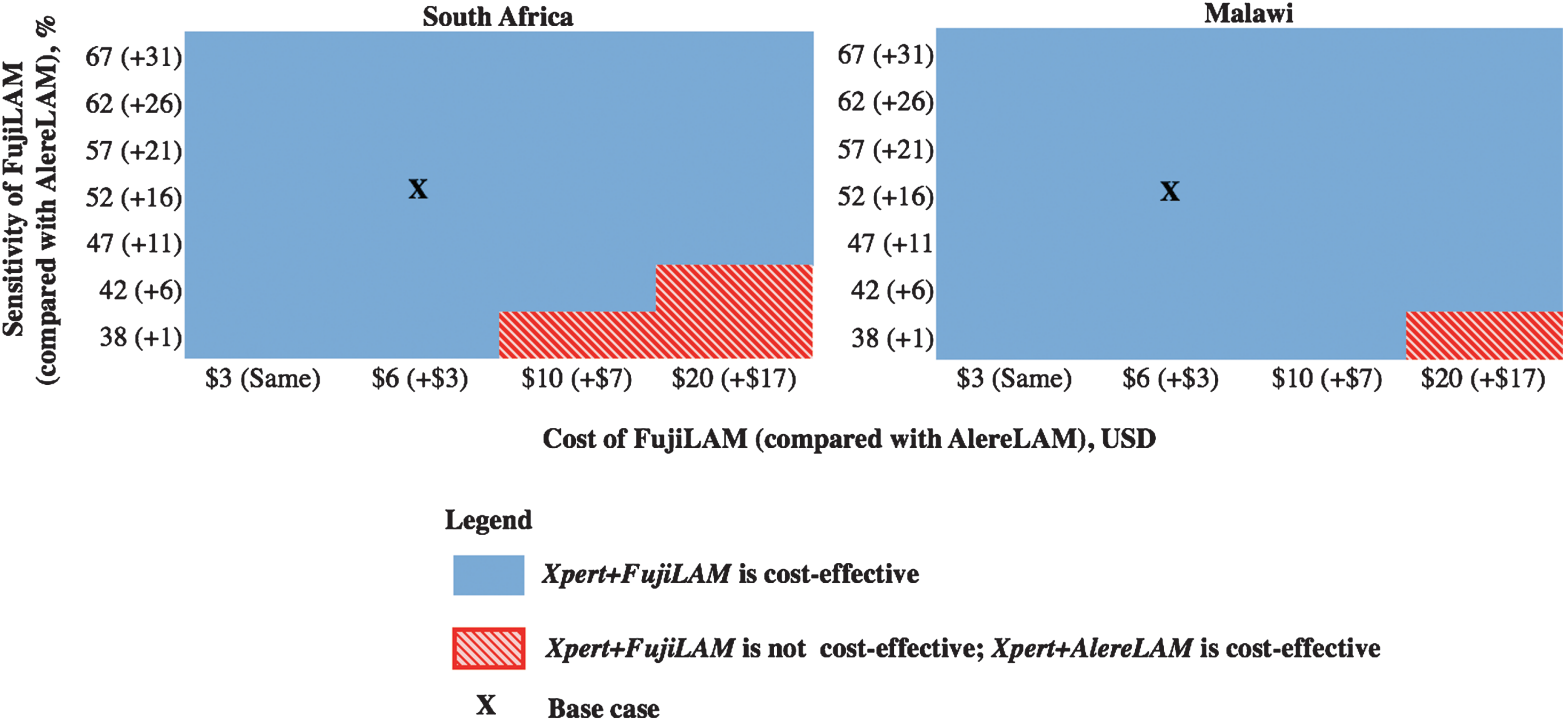
Two-way sensitivity analysis of FujiLAM sensitivity and cost. We varied FujiLAM sensitivity and FujiLAM per-test cost across ranges and compared the cost-effectiveness of *Xpert*, *Xpert+AlereLAM*, and *Xpert+FujiLAM*. The displayed sensitivities are weighted averages of the sensitivities among those with a CD4 count <200 cells/µL and ≥200 cells/µL. The numbers in parentheses show the difference in sensitivity between FujiLAM and AlereLAM. In the blue areas, *Xpert+FujiLAM* is cost-effective compared with both *Xpert* and *Xpert+AlereLAM*; it weakly dominates *Xpert+AlereLAM*, meaning that it is more effective and has a lower cost-effectiveness ratio than *Xpert+AlereLAM*. In the red hatched areas, *Xpert+FujiLAM* is not cost-effective compared with *Xpert*, but *Xpert+AlereLAM* is cost-effective compared with *Xpert*. In the base case in both countries, FujiLAM is 15% more sensitive than AlereLAM and costs USD 3 more per test. Abbreviations: LAM, lipoarabinomannan; USD, 2017 US dollars.

In multi-way deterministic sensitivity analyses in which we varied TB prevalence, sputum provision, and empiric TB treatment probability, *Xpert+FujiLAM* was cost-effective compared with *Xpert* in South Africa except when both TB prevalence was relatively low (15%) and sputum provision probability was high (90%); *Xpert+FujiLAM* was cost-effective compared with *Xpert* in Malawi in all scenarios (Supplementary Figure 2). In a 2-way sensitivity analysis where FujiLAM sensitivity and FujiLAM cost were varied, *Xpert+FujiLAM* remained cost-effective compared with *Xpert* in South Africa and Malawi, except when FujiLAM had both relatively low sensitivity (≤42%) and higher cost (≥USD 10/test) (Figure 1).

In the probabilistic sensitivity analysis, there was a more than 95% probability that *Xpert+FujiLAM* offered the highest net monetary benefit when willingness-to-pay was more than USD 930/YLS in South Africa and more than USD 460/YLS in Malawi (Supplementary Figure 3).

### Alternative Testing Strategies

**Figure 2.**
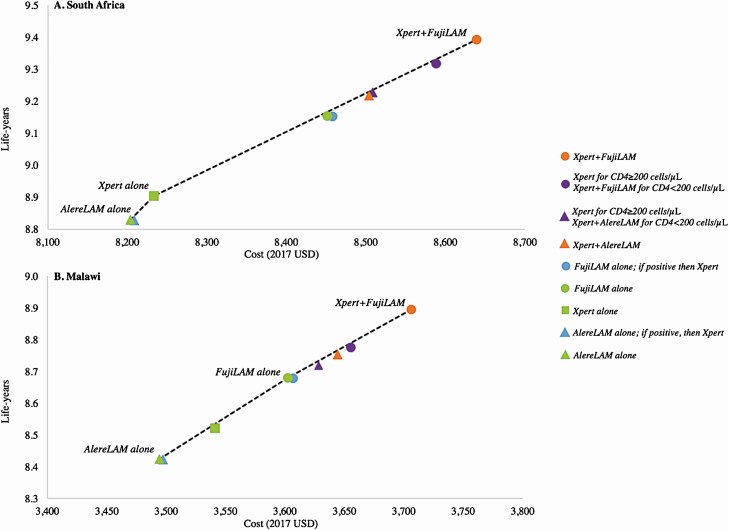
Cost-effectiveness frontier of alternative tuberculosis testing strategies in hospitalized people with HIV. We projected the life-years and lifetime costs associated with solo (green), parallel (orange), sequential (blue), and CD4-stratified (purple) tuberculosis testing strategies in South Africa (*A*) and Malawi (*B*). Squares represent a strategy of Xpert alone, triangles represent strategies that include AlereLAM, and circles represent strategies that include FujiLAM. The testing strategies labelled on the cost-effectiveness frontier line are those that were not dominated. Other strategies, represented by symbols below the line, were dominated, reflecting an inefficient use of resources. Abbreviations: HIV, human immunodeficiency virus; LAM, lipoarabinomannan; USD, 2017 US dollars.

Most solo, sequential, and CD4-stratified testing strategies were dominated by *Xpert+FujiLAM* (Supplementary Table 4). In South Africa, only *AlereLAM alone*, *Xpert alone*, and *Xpert+FujiLAM* were on the cost-effectiveness efficiency frontier (strategies below the frontier are dominated) (Figure 2A). In Malawi, only *AlereLAM alone*, *FujiLAM alone*, and *Xpert+FujiLAM* were on the efficiency frontier (Figure 2B). Strategies that added LAM testing to Xpert provided notable gains in life expectancy at modest additional cost compared with *Xpert alone*, the more established strategy. *Xpert+FujiLAM* provided the most life-years in both countries. Strategies that included Xpert Ultra instead of Xpert reduced 2-year mortality modestly (<0.8%); cost-effectiveness results were similar to those of the base case (Supplementary Table 5).

### Budget Impact Analysis

**Figure 3.**
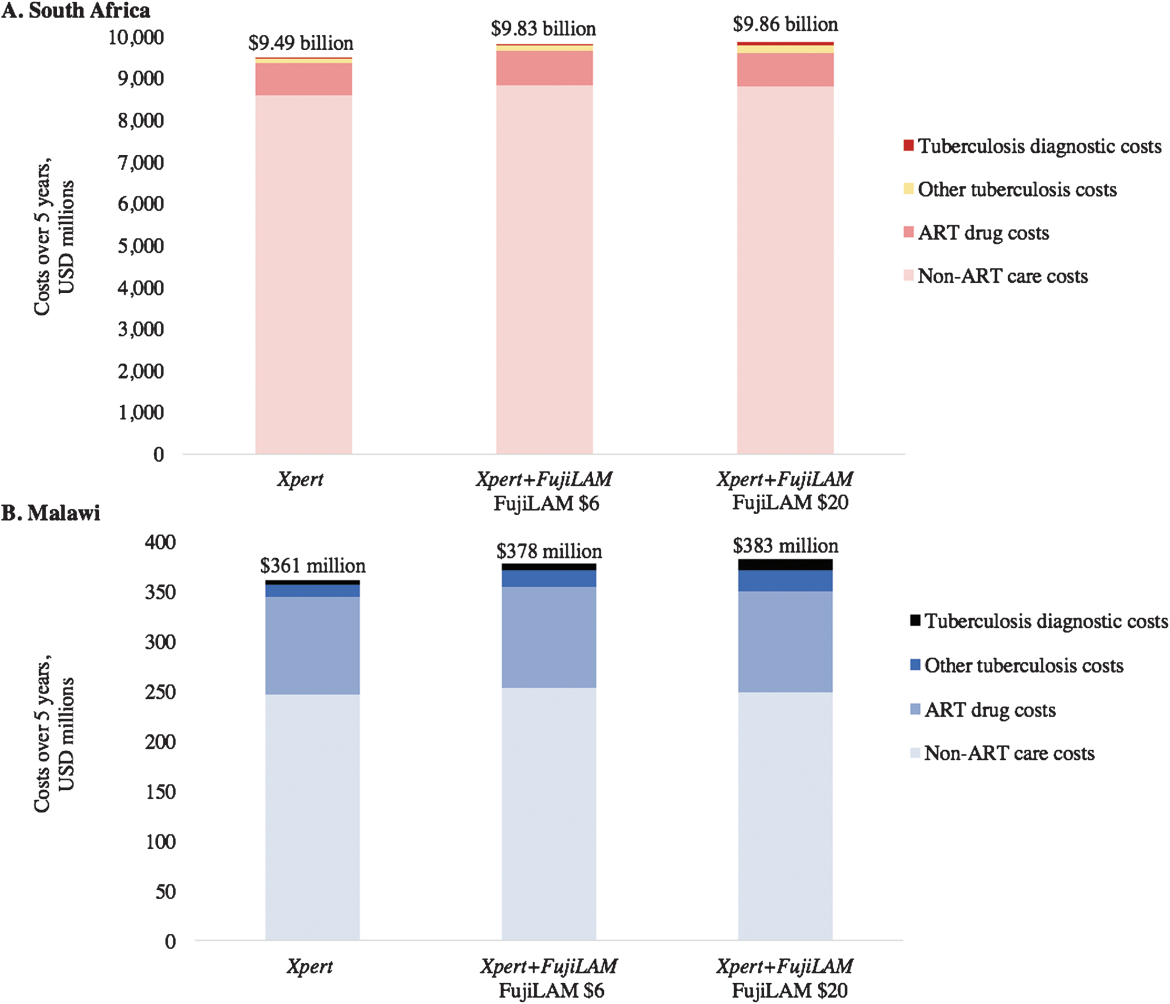
Budget impact analysis at 5-year horizon: implementing FujiLAM testing countrywide in South Africa (*A*) and Malawi (*B*) among hospitalized patients with HIV. The vertical axis range is different between panel *A* and panel *B*. Budgetary projections are for the estimated 500 000 people with HIV who would be hospitalized each year in South Africa and 70 000 people with HIV who would be hospitalized each year in Malawi, all of whom would undergo tuberculosis testing. Within each panel, the left bar represents 5-year cumulative healthcare costs among these people if *Xpert* was the tuberculosis testing strategy. The middle bar reflects the *Xpert+FujiLAM* testing strategy, with FujiLAM costing USD 6 per test. The right bar reflects the *Xpert+FujiLAM* testing strategy, with FujiLAM costing USD 20 per test. Abbreviations: ART, antiretroviral therapy; HIV, human immunodeficiency virus; LAM, lipoarabinomannan; USD, 2017 US dollars.

Over 5 years, testing all hospitalized PWH for TB with *Xpert+FujiLAM* instead of *Xpert* saved approximately 172 200 and 26 700 years of life in South Africa and Malawi, respectively. When FujiLAM per-test cost was USD 6, *Xpert+FujiLAM* increased cumulative healthcare expenditures among tested individuals by approximately USD 336 million (3.5%) in South Africa and USD 17 million (4.7%) in Malawi over 5 years, compared with *Xpert* (Figure 3). The largest contributors to the increase were non-TB, non-ART HIV care costs (70%/40% of increase in South Africa/Malawi). When excluding HIV care costs, *Xpert+FujiLAM* compared with *Xpert* increased 5-year TB healthcare expenditures among tested individuals by approximately USD 56 million (46%) in South Africa and USD 7 million (40%) in Malawi. FujiLAM itself, at USD 6 per test, contributed USD 15 million (South Africa) and USD 2 million (Malawi) to these additional costs. When FujiLAM per-test cost was USD 20, the increases in cumulative healthcare expenditures for both TB and HIV care were USD 370 million (3.9%) in South Africa and USD 22 million (6.1%) in Malawi. One-year budget impact results are shown in the Supplementary Text and Supplementary Figure 4.

## DISCUSSION

In our model-based analysis, we found that testing hospitalized PWH for TB with sputum Xpert and urine FujiLAM together decreased mortality, increased life expectancy by 0.6–0.7 years, and was cost-effective compared with sputum Xpert testing alone in South Africa and Malawi. A testing strategy of Xpert plus FujiLAM outperformed and economically dominated an Xpert plus AlereLAM strategy. The results remained robust in sensitivity analysis in which key parameters were varied to reflect other possible settings and scenarios. A novel aspect of this analysis was our comparison of clinically relevant parallel, solo, sequential, and CD4-stratified testing strategies—Xpert plus FujiLAM for all remained cost-effective.

In 2019, the WHO expanded its recommendations for AlereLAM use for TB diagnosis—for inpatient PWH, the WHO strongly recommends AlereLAM for those with signs and symptoms of TB, those with advanced HIV disease or who are seriously ill, and those with a CD4 count of less than 200 cells/µL, irrespective of signs and symptoms [24]. With its improved sensitivity, FujiLAM might be considered for broader use [6–8]. However, prospective studies to demonstrate feasibility, clinical outcomes, and cost in clinical practice settings will be important, as FujiLAM compared with AlereLAM requires additional steps (silver amplification) and time (50–60 minutes vs 25 minutes, including incubation) [6]. While we attempted to capture these operational factors by increasing the cost of FujiLAM, they are challenging to account for in cost-effectiveness analysis. Operational variability could influence FujiLAM accuracy and uptake but is unlikely to prolong time to treatment initiation after a positive result.

The per-test price of FujiLAM has not been finalized, and there are no published micro-costing estimates of FujiLAM in practice. A preliminary cost has been estimated at USD 6 per test, in line with WHO’s target for a new TB diagnostic [22]. Our sensitivity analysis showed that, even at a higher per-test cost, a testing strategy combining FujiLAM with sputum Xpert would be cost-effective compared with Xpert alone. Increasing the FujiLAM cost has little influence on the ICER—indeed, most incremental costs of FujiLAM strategies reflect years of HIV care for individuals who otherwise would die of undiagnosed TB.

However, FujiLAM cost has a greater influence in the budget impact analysis, which accounts for the total number of people who would be tested. Although we did not fully account for the operational factors associated with implementation and scale-up of FujiLAM testing or for the logistics of increasing TB treatment capacity, our budget impact analysis of FujiLAM at a cost of USD 20 per test indirectly reflects some of these factors by incorporating operational costs into the test cost. We show that adding FujiLAM would contribute a relatively small amount to the total healthcare costs for this patient population, and that much of the increase in costs is due to downstream positive effects of longer survival and not due to the test itself. Nonetheless, when considered in the isolated context of a TB-control program, adding FujiLAM would consume a greater proportion of the program’s budget. Overall, FujiLAM offers clinical benefit and good value based on the ICER, but affordability must be considered in the context of budget and other resource constraints and the full costs of implementation.

There is no consensus on appropriate cost-effectiveness thresholds in a given country [16]. As in a prior study, we used as our cost-effectiveness threshold the ICER of second-line ART, which is recommended for care by national guidelines in both South Africa and Malawi [9, 14, 15]. Alternative thresholds could affect interpretations of cost-effectiveness but would not change the model-generated ICER results.

Urine LAM assays are more sensitive in those with low CD4 counts compared with high CD4 counts [6]. Nonetheless, our analysis of CD4-stratified testing strategies showed that adding FujiLAM testing for all patients, rather than only for those with CD4 counts less than 200 cells/µL, would provide greater clinical benefit and be cost-effective. As CD4 testing for the diagnostic algorithm would add time delay, cost, and complexity (and it is being phased out in many settings), performing FujiLAM for all hospitalized PWH rather than only for those with low CD4 counts offers practical advantages.

We previously conducted a cost-effectiveness analysis of the STAMP trial, finding that adding AlereLAM to Xpert was cost-effective compared with Xpert alone [9]. In the present study, *Xpert+AlereLAM* remained cost-effective compared with *Xpert*, but *Xpert+FujiLAM* was cost-effective compared with *Xpert* and *Xpert+AlereLAM* and yielded higher life expectancy than those strategies. Compared with the prior study’s results, we project fewer life-years in South Africa and a smaller difference in life-years between testing strategies in Malawi. These discrepancies are due to differences in diagnostic yields and other model parameters between the 2 studies. In our previous study, we applied parameters directly from the STAMP trial in which there were differences between South Africa and Malawi, including a much higher probability of sputum provision (75% vs 39%), higher probability of empiric treatment (10% vs 4%), and lower incremental diagnostic yield of AlereLAM (~19% vs 52%) in South Africa compared with Malawi [5]. We assumed here that sputum provision probability, empiric treatment probability, and diagnostic yield would be similar in the 2 countries. Despite modest changes in these assumptions, both studies showed that adding LAM to Xpert would be cost-effective, and our sensitivity analyses in this study (which included the base case values from the STAMP cost-effectiveness analysis) provide insight into results when parameters differ by country. Additional testing of urine by Xpert, as in the STAMP trial, could be considered. However, urine Xpert had only limited additional diagnostic yield above urine AlereLAM and sputum Xpert in STAMP, and it had disadvantages of requiring centrifugation and costing more than AlereLAM [5, 9]. Xpert Ultra may offer greater yield.

Like all model-based analyses, our study has limitations. We applied diagnostic yields from a retrospective study (with the attendant potential biases) that included all assays of interest, except for applying a base case sputum provision probability of 50% [5, 6, 17, 25, 26]. We chose this retrospective study because it included helpful details about the additional diagnostic yields of AlereLAM and FujiLAM above sputum Xpert alone [6]. Our analysis accounted for false-positive test results in terms of costs of unnecessary TB treatment and of managing treatment toxicities but did not account for potential mortality from misdiagnosis and unnecessary treatment, which could temper the enthusiasm for more widespread implementation (although, because of an imperfect reference standard, some “false-positives” may be true-positives). Lacking data, we did not account for TB transmission, thus potentially underestimating population-level benefits of LAM testing in detecting TB, prompting treatment, and decreasing transmission. Finally, we adopted a health system perspective for costs and did not include patient costs, non–health system costs, or economic gains from improved survival.

In conclusion, our model-based analysis found that adding urine FujiLAM to sputum Xpert for TB testing among unselected hospitalized PWH in South Africa and Malawi would increase life expectancy and be cost-effective. Although additional feasibility studies of FujiLAM are needed in clinical practice settings, the rapidity of the test procedure and its improved sensitivity over an earlier-generation LAM assay suggest that it would reduce deaths among hospitalized PWH in TB-endemic settings while offering good value when its cost is in line with WHO targets.

## Supplementary Data

Supplementary materials are available at *Clinical Infectious Diseases* online. Consisting of data provided by the authors to benefit the reader, the posted materials are not copyedited and are the sole responsibility of the authors, so questions or comments should be addressed to the corresponding author.

ciaa1698_suppl_Supplementary_Materials_1

## Notes

***Author contributions.*** K. P. R., C. M. D., T. B., and R. P. W. conceived the study. K. P. R., N. C. M., K. A. F., and R. P. W. developed the model structure and designed the cost-effectiveness analysis. K. P. R., N. C. M., P. P. P., F. M. S., C. R. H., and R. W. developed model input parameters. C. M. D., T. B., A. G.-W., A. D. K., K. L. F., M. P. N., and G. M. collected and analyzed primary trial data for use in the model. K. P. R. and N. C. M. implemented the model and analyzed the data. K. P. R. wrote the first manuscript draft. All authors contributed to the study design and interpretation of the results, revised the manuscript for important intellectual content, and approved the final version.

***Financial support.*** This work was supported by awards from the National Institute of Allergy and Infectious Diseases (grant numbers R37 AI093269, R37 AI058736) of the National Institutes of Health; Massachusetts General Hospital (Steve and Deborah Gorlin Research Scholars Award to R. P. W.); and the Joint Global Health Trials Scheme of the UK Department of Health and Social Care, the Department for International Development, the Global Challenges Research Fund, the Medical Research Council, and Wellcome Trust (grant number MR/M007375/1), a UK-funded award that is part of the EDCTP2 program supported by the European Union. C. R. H. is also supported by the Providence/Boston Center for AIDS Research (grant number P30 AI042853 from the National Institute of Allergy and Infectious Diseases of the National Institutes of Health) and the Boston University/Rutgers Tuberculosis Research Unit (grant number U19 AI111276 from the National Institute of Allergy and Infectious Diseases of the National Institutes of Health). G. M. was supported by the Wellcome Trust (grant numbers 098316, 203135/Z/16/Z), the South African Research Chairs Initiative of the Department of Science and Technology and National Research Foundation (NRF) of South Africa (grant number 64787), NRF incentive funding (UID: 85858), and the South African Medical Research Council through its TB and HIV Collaborating Centres Programme with funds received from the National Department of Health (RFA# SAMRC-RFA-CC: TB/HIV/AIDS-01-2014). M. P. N. reports grants from the Foundation for Innovative New Diagnostics during the conduct of the study. K. L. F. reports grants from Global Health Trials (MRC/DFID/WT) during the conduct of the study. C. M. D. and T. B. report grants for the development and evaluation of FujiLAM (not directly for this modeling work) from UK Department for International Development, German Federal Ministry of Education and Research (BMBF), through Kreditanstalt für Wiederaufbau, the Dutch Ministry of Foreign Affairs, Australian Department of Foreign Affairs and Trade, and Global Health Innovative Technology, during the conduct of the study.

***Potential conflicts of interest.*** C. M. D. and T. B. were previously employed by Foundation for Innovative Diagnostics (FIND). FIND is a not-for-profit foundation that supports the evaluation of publicly prioritized tuberculosis assays and the implementation of WHO-approved (guidance and prequalification) assays using donor grants. FIND has product evaluation agreements with several private sector companies that design diagnostics for tuberculosis and other diseases. These agreements strictly define FIND’s independence and neutrality vis-à-vis the companies whose products get evaluated and describe roles and responsibilities. T. B. reports a patent-pending (WO/2019/186486) in the field of lipoarabinomannan detection, as an only inventor, but without ownership/commercial rights. All other author report no potential conflicts. All authors have submitted the ICMJE Form for Disclosure of Potential Conflicts of Interest. Conflicts that the editors consider relevant to the content of the manuscript have been disclosed.
