# Supplementary material for: Cost-effectiveness of a Novel Lipoarabinomannan Test for Tuberculosis in Patients With Human Immunodeficiency Virus
Source: Clin Infect Dis. 2020 Nov 17;73(7):e2077–85. doi: 10.1093/cid/ciaa1698 (PMC8492225; doi:10.1093/cid/ciaa1698)
Supplement: ciaa1698_suppl_Supplementary_Materials_1 [file ciaa1698_suppl_supplementary_materials_1.docx]

**SUPPLEMENT**

**Cost-effectiveness of a novel lipoarabinomannan test for tuberculosis in patients with HIV**

Krishna P. Reddy, Claudia M. Denkinger, Tobias Broger, Nicole C. McCann, Ankur Gupta-Wright,

Andrew D. Kerkhoff, Pamela P. Pei, Fatma M. Shebl, Katherine L. Fielding, Mark P. Nicol,

C. Robert Horsburgh, Graeme Meintjes, Kenneth A. Freedberg, Robin Wood,

Rochelle P. Walensky

**Contents**

Methods: Additional Information p. 2 1. Analytic Overview 2

2. Model Overview 3

3. Diagnostic Yield Calculations 5

4. Model Calibration and Validation 7

5. Input Parameters 8

6. Deterministic Sensitivity Analysis 10

7. Probabilistic Sensitivity Analysis 11

8. Budget Impact Analysis 12

Results: Additional Information 14

Sensitivity Analysis: Dolutegravir-based ART 14

Budget Impact Analysis at One Year 14

Supplementary References 15

Supplementary Table 1 20

Supplementary Table 2 21

Supplementary Table 3 22

Supplementary Table 4 29

Supplementary Table 5 31

Supplementary Figure 1 33

Supplementary Figure 2 35

Supplementary Figure 3 37

Supplementary Figure 4 39

**METHODS: ADDITIONAL INFORMATION**

**1. Analytic Overview**

To evaluate tuberculosis (TB) testing strategies, we simulated cohorts of one million hospitalized people with HIV (PWH) in either South Africa or Malawi in the Cost-Effectiveness of Preventing AIDS Complications (CEPAC)-International microsimulation model ([massgeneral.org/medicine/mpec/research/cpac-model](http://www.massgeneral.org/medicine/mpec/research/cpac-model)). We followed each simulated patient over her/his lifetime and counted all years of life and TB and HIV care costs accrued during that lifetime. The model is coded in C++. As a microsimulation model, CEPAC-International has “memory” of past events and enables the simulation of many different combinations of patient characteristics and health states; the number of these would be unwieldy to simulate in a traditional Markov model.

As in our prior cost-effectiveness analysis of TB testing strategies among PWH in South Africa and Malawi, we used the incremental cost-effectiveness ratio (ICER) of second-line antiretroviral therapy (ART) as the cost-effectiveness threshold [1]. This reflects a “benchmark intervention” approach to determining a cost-effectiveness threshold – second-line ART is relatively expensive but is implemented and recommended in national HIV care guidelines in both South Africa and Malawi [2–4]. We previously used the CEPAC-International model to determine the ICER of a care strategy that includes second-line antiretroviral therapy (ART; for those who fail first-line ART) to a strategy that does not include second-line ART. This ICER, which we used as the cost-effectiveness threshold, was USD940 per year-of-life saved (YLS) in South Africa and USD750/YLS in Malawi [1]. We report both undiscounted and discounted (3%/year) outcomes; we used the latter in cost-effectiveness analysis [5].

**2. Model Overview**

Tuberculosis Natural History and Diagnosis

In any given month, simulated individuals are in one of several possible TB states: Uninfected, Latent, Active, Previously Treated, or Lost to Follow-up from Treatment (Supplementary Figure 1). Probabilities of transition between these TB states depend on probabilities of infection, reactivation, treatment success, relapse, and loss to follow-up (LTFU). The TB state in the model reflects the true status of the patient and not the clinically observed status.

Throughout a lifetime, a simulated patient has a probability of developing TB, either for the first time or as a recurrence from relapse or reinfection. The user defines monthly probabilities of infection (and reinfection), after which there is a CD4-dependent monthly probability of reactivation. In addition, the user defines a time-dependent monthly probability of relapse after an episode of TB.

Tuberculosis Treatment

After diagnosis, individuals start treatment for drug-susceptible TB (in case of no result or negative result for rifampicin resistance from sputum Xpert) or multidrug-resistant TB (in case of positive result for rifampicin resistance from sputum Xpert). Those whose only positive result is by urine AlereLAM or FujiLAM are treated for drug-susceptible TB and do not undergo additional drug-susceptibility testing, reflecting a lack of sputum specimen or a negative sputum Xpert result and the reality of clinical decision making in these contexts.

Each TB treatment regimen is associated with a probability of success (interpreted as cure, or negative culture at end of treatment), which depends on the efficacy of the regimen for the underlying TB strain (e.g., DS or MDR). When an individual starts a TB treatment regimen, the model assigns either “successful” or “failed” treatment, based on a random draw from the defined probability distribution. Those assigned to “success” do not die from TB while on treatment, and they move from the Active TB state to the Previously Treated state upon completing treatment. Those assigned to “failed” treatment remain in the Active TB state and are subject to a monthly TB mortality probability.

Loss to Follow-up and Non-adherence to Treatment

After hospital discharge, individuals are subject to a monthly probability of LTFU from TB care, wherein they stop taking TB treatment. At that time, those who had been initially assigned to treatment “success” again draw randomly for a probability of success, which we assume is dependent on the proportion of treatment duration they have completed (e.g., those who are lost to follow-up and stop taking treatment four months into a planned six-month course now have a 4/6 probability of having been successfully treated and cured) [6]. Those who are again assigned “successful” treatment transition to the Lost to Follow-up from Treatment state and can face a higher risk of TB relapse and acquired drug resistance compared with those who completed a full course of treatment. Those who are assigned “failed” treatment remain in the Active TB state and are subject to a monthly TB mortality probability.

HIV Treatment

Simulated individuals not already on ART are eligible for immediate ART initiation, regardless of CD4 count, with tenofovir/lamivudine/efavirenz, the most common first-line regimen recommended in both Malawi and South Africa [2,3]. The efficacy of the ART regimen (the probability of viral suppression) is related to an individual’s adherence. Those who adhere to efficacious ART experience a decrease in HIV RNA, an increase in CD4 count, and a decrease in HIV-related morbidity and mortality [7]. Those whose HIV is initially suppressed face a regimen-specific monthly probability of treatment failure after 48 weeks; this probability is inversely correlated with adherence to ART. Treatment failure is detected according to country-specific guidelines [2,8]. Those who fail first-line treatment are eligible to receive a second-line, protease inhibitor-based ART regimen. If drug toxicity is noted, individuals can be switched to an alternative ART regimen. Individuals also have a monthly probability of LTFU from HIV care, distinct from the probability of LTFU from TB care (per expert opinion of colleagues practicing in Africa), wherein they completely discontinue ART [7]. After LTFU from HIV care, there is a monthly probability of returning to HIV care. Additional specifications about HIV care are described elsewhere [9,10].

Causes of Death

Causes of death in the model include TB, another opportunistic infection/disease, chronic HIV, and conditions not directly caused by HIV. Specific opportunistic infections/diseases are *Cryptococcus* meningitis, toxoplasmosis, *Pneumocystis jiroveci* pneumonia, atypical mycobacterial infection, cytomegalovirus infection, severe bacterial infection, and lymphoma. Chronic HIV-related mortality is that due to classic HIV syndromes and disorders, such as chronic wasting, that are not directly caused by an opportunistic infection. Incidence and mortality rates, converted to monthly probabilities for use in the model, are derived from the literature and are described elsewhere ([massgeneral.org/medicine/mpec/research/cpac-model](http://www.massgeneral.org/medicine/mpec/research/cpac-model)) [9–12].

**3. Diagnostic Yield Calculations**

We calculated the diagnostic yields of *Xpert+AlereLAM* and *Xpert+FujiLAM* based on data from Broger et al. [13]. We defined diagnostic yield as the number of tuberculosis cases correctly identified by a testing strategy (accounting for the ability to obtain a sample) divided by the number of true tuberculosis cases according to a study-defined composite reference standard [13].

When varying sputum provision probability, we adjusted the diagnostic yield of the Xpert+LAM strategies to account for the additional or fewer cases that would be detected by Xpert. One correction involved adjusting the *incremental* diagnostic yield – for example, when increasing sputum provision probability, some of the additional cases now detected by sputum Xpert were already detected by LAM and therefore would not increase the overall diagnostic yield of an Xpert+LAM strategy.

We assumed that the ratio of the number of cases detected only by Xpert (and not by LAM) to the total number of cases detected by Xpert remained constant as sputum provision changed. For example, based on data from Broger et al., in which the sputum provision probability was 30%, sputum Xpert identified 36 cases of tuberculosis [13]. Of these 36, 10 (28%) were detected by sputum Xpert and not by FujiLAM, and 26 were detected by both sputum Xpert and urine FujiLAM. Urine FujiLAM detected 91 cases, including those also detected by sputum Xpert. When varying sputum provision probability, we calculated the diagnostic yield by adding the proportion of cases detected by FujiLAM to the following product: sputum provision probability [varies] * Xpert sensitivity [0.65] * ratio of the number of cases detected only by Xpert (and not by FujiLAM) to the total number of cases detected by Xpert [0.28].

When varying FujiLAM sensitivity, we assumed that the incremental yield of sputum Xpert also varied in the *Xpert+FujiLAM* strategy. For example, we assumed that as FujiLAM sensitivity decreased, the incremental yield of sputum Xpert increased. In the study by Broger et al., the incremental yield of sputum Xpert to AlereLAM was higher than that of Xpert to FujiLAM [13]. AlereLAM and FujiLAM provided two empiric data points to determine the relationship between LAM sensitivity and the incremental yield of Xpert. We derived a linear equation that fit these data points: 1) the incremental yield of sputum Xpert to AlereLAM when AlereLAM sensitivity was at the base case value; 2) the incremental yield of sputum Xpert to FujiLAM when FujiLAM sensitivity was at the base case value. We then linearly interpolated the incremental yield of Xpert over FujiLAM at any given FujiLAM sensitivity.

Xpert Ultra has higher sensitivity than Xpert for TB detection (Table 1 in main text).[14] Diagnostic yields of strategies that included Xpert Ultra were as follows (assuming 50% sputum provision probability): *XpertUltra*, 39%; *XpertUltra+AlereLAM*, 65% for those with CD4<200/µL and 41% for those with CD4≥200/µL; *XpertUltra+FujiLAM*, 71% for those with CD4<200/µL and 52% for those with CD4≥200/µL.

**4. Model Calibration and Validation**

We previously performed internal validation of the model by calibrating mortality probabilities to match all-cause mortality at two months reported in the STAMP trial [1,15]. We performed calibrations separately by country, by group (intervention or control), and by CD4 count (<200/µL or ≥200/µL, new for this analysis). Of note, among STAMP trial participants in South Africa with CD4≥200/µL, two-month mortality was higher among those in the intervention group (sputum Xpert + urine Xpert + urine LAM) compared with the standard of care group (sputum Xpert). Though the difference was small and likely due to chance, for consistency we calibrated mortality in our model to match these mortality probabilities.

For external and longer-term validation, we previously simulated ambulatory PWH initiating ART in South Africa and compared model-generated longer-term mortality results to those reported in South African cohort studies [1,16–19].

**5. Input Parameters**

Tuberculosis Prevalence

As described in our previous study, we derived underlying TB prevalence (29% in South Africa and 24% in Malawi) using STAMP trial data, consistent with the prevalence reported in other studies [1,20–23]. Based on rifampicin resistance data among STAMP participants, we assumed 3% (South Africa) and 1% (Malawi) of those with TB had multidrug-resistant disease [15].

Tuberculosis Diagnostics

We calculated the sensitivity of each test as: among those who provided a specimen for a test (i.e., sputum for Xpert), the number of subjects who had a correct positive result by that test, divided by the number of subjects diagnosed with TB according to a composite reference standard (Table 1) [13]. The composite reference standard included: 1) microbiologically confirmed TB (by any positive culture or Xpert result); or 2) clinical or radiological features suggestive of TB and initiation of TB treatment. Sensitivity calculations did not include those unable to provide a specimen.

Sputum specimen provision was ~30% among subjects in the Broger et al. study, whereas it was 28-75% in other studies of LAM in hospitalized PWH [13,15,22,24,25]. Given the findings in other studies, and based on expert opinion, in our base case we assumed that 50% of individuals would provide a sputum specimen. Therefore, to calculate the diagnostic yield of *Xpert* in the base case, we multiplied the sensitivity of sputum Xpert by 0.5. To calculate the diagnostic yield of *Xpert+AlereLAM* and *Xpert+FujiLAM* in the base case, we again assumed that 50% of individuals would provide a sputum specimen.

Tuberculosis Treatment and Natural History

We assumed in the base case that empiric treatment would be given within the first month of model simulation to 11% of individuals in the *Xpert* strategy and 10% of individuals in the *Xpert+AlereLAM* or *Xpert+FujiLAM* strategies [13,15]. We assumed the likelihood of empiric treatment among those who truly had TB would be double that among those who did not have TB. We parameterized TB treatment outcomes, including regimen efficacies, based on data reported in prior studies (Supplementary Table 1). We assumed that those who survived for two years with untreated TB self-cured, such that they transitioned to the Previously Treated state despite not having received treatment.

Individuals on TB treatment have a monthly probability of experiencing toxicity, which is associated with a cost but not with mortality [26,27]. Additional input parameters are in Table 1 and Supplementary Table 1.

HIV Treatment and Loss to Follow-up

Among those initiating ART, 72% achieved virologic suppression at 48 weeks [28,29]. Additional HIV treatment parameters, including those around ART efficacy and toxicity, were applied as per published CEPAC-International studies in sub-Saharan Africa [9,10]. After the initial hospitalization, the probability of LTFU from HIV care ranged from 0.2% to 1.6% monthly, yielding a five-year probability of remaining in HIV care of 74% [30,31].

In a sensitivity analysis, we considered dolutegravir-based ART instead of efavirenz-based ART. Based on the Clinton Health Access Initiative reference price list, the cost of the dolutegravir-based regimen was $6.25 per month, compared with $11 per month for the efavirenz-based regimen [32,33]. The dolutegravir-based regimen had higher efficacy [34].

Resource Utilization and Costs

All costs were from the health system perspective. We applied hospital-based resource utilization and costs, apart from those for TB diagnostic testing, as described previously [1]. Only those who provided a sputum specimen accrued the cost of sputum Xpert testing.

We applied country-specific costs of HIV care, including ART and non-ART care (Supplementary Table 1). Monthly care costs did not differ by TB testing strategy, but because those who survived longer accrued more costs of chronic HIV care, TB testing strategies that increased life expectancy also increased HIV care costs.

Cumulative healthcare costs included: 1) initial hospitalization costs; 2) post-hospitalization TB costs (clinical visits, medications); 3) ART drug costs; 4) non-ART HIV care costs (clinical visits, CD4 testing, HIV RNA testing, subsequent hospitalizations). Key costs are shown in Table 1 and Supplementary Table 1.

We adjusted costs to 2017 US dollars, as described previously [1]. When Malawi costs were not available, we used purchasing power parity to convert South Africa costs to Malawi costs [35]. We assumed that drug costs were equal in South Africa and Malawi due to importation and ceiling prices for low-resource settings [36].

**6. Deterministic Sensitivity Analysis**

One of our deterministic sensitivity analyses involved varying FujiLAM sensitivity, which impacted the diagnostic yield of the *Xpert+FujiLAM* strategy. When increasing FujiLAM sensitivity, some of the cases newly detected by FujiLAM would have been already detected by sputum Xpert, and therefore the impact of higher FujiLAM sensitivity on *Xpert+FujiLAM* diagnostic yield is attenuated and less than a simple additive effect

**7. Probabilistic Sensitivity Analysis**

In a probabilistic sensitivity analysis (PSA), we simultaneously varied several parameters across beta distributions (Table 1). We used raw data when available to calculate α and β. When a PSA input was derived from multiple sources or adjusted in any way in the base case, we incorporated multiple distributions and adjusted the distribution accordingly. We first found the α and β for each component of the model input and then performed the necessary operation on the distributions to create the final PSA input parameter distribution. For example, in our base case analysis, we derived LTFU by using a weighted average of two published values [37,38]. For our PSA input distribution, we first found the α and β values from each published source, and then applied a weighted average of these distributions to yield a final distribution. When no raw data was available – for example in the case of sputum provision probability which was an assumption in the base case – we used the Microsoft Excel beta distribution function to create the beta distribution.

The α/β values were: sputum provision probability, 3/3; probability of empiric TB treatment for those in the *Xpert* strategy, 70/557; probability of empiric TB treatment for those in the *Xpert+AlereLAM* and *Xpert+FujiLAM* strategies, 61/570; monthly probability of death from untreated TB, 9.3/98.6. Similar to the methods in a prior study, we estimated a distribution for TB prevalence based on the equation: 1.25 * (number of microbiologically confirmed TB cases with the *Xpert+FujiLAM* strategy, α=95, β=536) + (number of clinically diagnosed TB cases, α=61, β=570) [1,39]. We estimated a distribution for monthly LTFU from TB care based on weighted data from two studies, each of which had a beta distribution (value 1, α=11, β=85; value 2, α=4, β=40.4).[37,38] Taken together, the 2.5^th^-97.5^th^ percentiles for the values drawn from the beta distributions were approximately: sputum provision probability, 15-84%; probability of empiric treatment for those in the *Xpert* strategy, 9-14%; probability of empiric treatment for those in the *Xpert+AlereLAM* and *Xpert+FujiLAM* strategies, 7-11%; monthly probability of death from untreated TB, 4-14%; TB prevalence, 24-33%; monthly LTFU from TB care, 2-5%.

We performed 1,000 sets of draws, where each draw represented a value randomly chosen from the distribution of each parameter. For each set of draws, we simulated one million individuals. We used the results of the PSA to generate a cost-effectiveness acceptability curve (CEAC) for South Africa and Malawi. The CEAC shows the probability, at alternative willingness-to-pay thresholds, that a testing strategy would be preferred in terms of net monetary benefit (NMB); NMB is defined as (life-years)*(willingness-to-pay) – (lifetime costs) [40–42].

**8. Budget Impact Analysis**

We conducted a budget impact analysis of adding FujiLAM to sputum Xpert for TB testing among all hospitalized adult PWH in South Africa and Malawi countrywide over one-year and five-year periods. We assumed that in South Africa there would be approximately 500,000 hospitalizations of PWH per year, based on estimates of 422 public hospitals and mean of 1,219 HIV/AIDS-related admissions per public hospital [43–45]. For Malawi, we scaled the 500,000 hospitalizations estimate from South Africa by the ratio of the number of adults with HIV in South Africa and Malawi (6.8 million and 0.92 million) [46]. This provided an estimate of approximately 70,000 hospitalizations of PWH per year in Malawi.

We multiplied the model-generated per-person estimates of clinical and economic outcomes (life-years and costs) by the number of hospitalizations per year, under the simplifying assumption that each hospitalization represented a different person. For the five-year budget impact analysis, there were new hospitalizations each year (500,000/year in South Africa and 70,000/year in Malawi) that contributed to the overall five-year costs. We assumed that those hospitalized in the first year would accumulate five years of life and costs, unless they died earlier; those hospitalized in the second year would accumulate four years of life and costs, unless they died earlier, etc.

**RESULTS: ADDITIONAL INFORMATION**

**Sensitivity Analysis: Dolutegravir-based ART**

When the first-line ART regimen was dolutegravir-based rather than efavirenz-based, life expectancy increased across all strategies by approximately 1.0-1.1 discounted life-years in South Africa and Malawi, and lifetime discounted costs decreased by approximately USD110-150. *Xpert+FujiLAM* remained cost-effective compared with *Xpert* and *Xpert+AlereLAM*, with ICER USD710/YLS in South Africa and USD380/YLS in Malawi.

**Budget Impact Analysis at One Year**

Over one year, testing all hospitalized PWH for TB with *Xpert+FujiLAM* instead of *Xpert* saved approximately 11,780 years of life in South Africa and 1,610 years of life in Malawi. When FujiLAM per-test cost was USD6, *Xpert+FujiLAM* increased cumulative healthcare expenditures among tested individuals by approximately USD29.6million (2.1%) in South Africa and USD1.7 million (4.4%) in Malawi over one year, compared with *Xpert* (Supplementary Figure 4). The largest contributors to the increase in costs were non-TB, non-ART HIV care costs in South Africa (55%) and TB-related, non-diagnostic costs in Malawi (52%). When looking only at TB and not HIV care costs, *Xpert+FujiLAM* compared with *Xpert* increased one-year TB healthcare expenditures among tested individuals by approximately USD11.1 million (48%) in South Africa and USD1.3 million (40%) in Malawi. The FujiLAM test itself, at a cost of USD6 per test, contributed USD3.0 million (South Africa) and USD0.4 million (Malawi) to these additional costs. When FujiLAM per-test cost was USD20, the increases in cumulative healthcare expenditures (for both TB and HIV care) were USD36 million (2.6%) in South Africa and USD2.6 million (7.0%) in Malawi.

**SUPPLEMENTARY REFERENCES**

1. Reddy KP, Gupta-Wright A, Fielding KL, et al. Cost-effectiveness of urine-based tuberculosis screening in hospitalised patients with HIV in Africa: a microsimulation modelling study. Lancet Glob Health **2019**; 7:e200–e208.

2. Ministry of Health, Malawi. Malawi Guidelines for Clinical Management of HIV in Children and Adults. 2016; Available at: https://aidsfree.usaid.gov/sites/default/files/malawi_art_2016.pdf.

3. Meintjes G, Moorhouse MA, Carmona S, et al. Adult antiretroviral therapy guidelines 2017. South Afr J HIV Med **2017**; 18:776.

4. Marseille E, Larson B, Kazi DS, Kahn JG, Rosen S. Thresholds for the cost-effectiveness of interventions: alternative approaches. Bull World Health Organ **2015**; 93:118–124.

5. Sanders GD, Neumann PJ, Basu A, et al. Recommendations for conduct, methodological practices, and reporting of cost-effectiveness analyses: Second Panel on Cost-Effectiveness in Health and Medicine. JAMA **2016**; 316:1093–1103.

6. Reddy KP, Horsburgh CR, Wood R, et al. Shortened tuberculosis treatment for people with HIV in South Africa: a model-based evaluation and cost-effectiveness analysis. Ann Am Thorac Soc **2020**; 17:202–211.

7. Ross EL, Weinstein MC, Schackman BR, et al. The clinical role and cost-effectiveness of long-acting antiretroviral therapy. Clin Infect Dis **2015**; 60:1102–1110.

8. Department of Health, Republic of South Africa. National Consolidated Guidelines for the Prevention of Mother-to-Child Transmission of HIV (PMTCT) and the Management of HIV in Children, Adolescents and Adults. 2015; Available at: http://www.sahivsoc.org/Files/ART%20Guidelines%2015052015.pdf. Accessed 28 April 2020.

9. Walensky RP, Ross EL, Kumarasamy N, et al. Cost-effectiveness of HIV treatment as prevention in serodiscordant couples. N Engl J Med **2013**; 369:1715–1725.

10. Walensky RP, Borre ED, Bekker L-G, et al. The anticipated clinical and economic effects of 90-90-90 in South Africa. Ann Intern Med **2016**; 165:325–333.

11. Holmes CB, Wood R, Badri M, et al. CD4 decline and incidence of opportunistic infections in Cape Town, South Africa: implications for prophylaxis and treatment. J Acquir Immune Defic Syndr **2006**; 42:464–469.

12. United Nations Department of Economic and Social Affairs. World Population Prospects, the 2008 Revision. Available at: http://www.un.org/en/development/desa/population/publications/trends/population-prospects.shtml. Accessed 28 April 2020.

13. Broger T, Sossen B, du Toit E, et al. Novel lipoarabinomannan point-of-care tuberculosis test for people with HIV: a diagnostic accuracy study. Lancet Infect Dis **2019**; 19:852–861.

14. Dorman SE, Schumacher SG, Alland D, et al. Xpert MTB/RIF Ultra for detection of Mycobacterium tuberculosis and rifampicin resistance: a prospective multicentre diagnostic accuracy study. Lancet Infect Dis **2018**; 18:76–84.

15. Gupta-Wright A, Corbett EL, van Oosterhout JJ, et al. Rapid urine-based screening for tuberculosis in HIV-positive patients admitted to hospital in Africa (STAMP): a pragmatic, multicentre, parallel-group, double-blind, randomised controlled trial. Lancet **2018**; 392:292–301.

16. Bassett IV, Coleman SM, Giddy J, et al. Barriers to care and 1-year mortality among newly diagnosed HIV-infected people in Durban, South Africa. J Acquir Immune Defic Syndr **2017**; 74:432–438.

17. Estill J, Egger M, Johnson LF, et al. Monitoring of antiretroviral therapy and mortality in HIV programmes in Malawi, South Africa and Zambia: mathematical modelling study. PLoS One **2013**; 8:e57611.

18. Boulle A, Schomaker M, May MT, et al. Mortality in patients with HIV-1 infection starting antiretroviral therapy in South Africa, Europe, or North America: a collaborative analysis of prospective studies. PLoS Med **2014**; 11:e1001718.

19. Cornell M, Johnson LF, Wood R, et al. Twelve-year mortality in adults initiating antiretroviral therapy in South Africa. J Int AIDS Soc **2017**; 20:21902.

20. Peter JG, Zijenah LS, Chanda D, et al. Effect on mortality of point-of-care, urine-based lipoarabinomannan testing to guide tuberculosis treatment initiation in HIV-positive hospital inpatients: a pragmatic, parallel-group, multicountry, open-label, randomised controlled trial. Lancet **2016**; 387:1187–1197.

21. Lawn SD, Kerkhoff AD, Burton R, et al. Rapid microbiological screening for tuberculosis in HIV-positive patients on the first day of acute hospital admission by systematic testing of urine samples using Xpert MTB/RIF: a prospective cohort in South Africa. BMC Med **2015**; 13:192.

22. Lawn SD, Kerkhoff AD, Burton R, et al. Diagnostic accuracy, incremental yield and prognostic value of Determine TB-LAM for routine diagnostic testing for tuberculosis in HIV-infected patients requiring acute hospital admission in South Africa: a prospective cohort. BMC Med **2017**; 15:67.

23. Ford N, Matteelli A, Shubber Z, et al. TB as a cause of hospitalization and in-hospital mortality among people living with HIV worldwide: a systematic review and meta-analysis. J Int AIDS Soc **2016**; 19:20714.

24. Boyles TH, Griesel R, Stewart A, Mendelson M, Maartens G. Incremental yield and cost of urine Determine TB-LAM and sputum induction in seriously ill adults with HIV. Int J Infect Dis **2018**; 75:67–73.

25. Huerga H, Ferlazzo G, Bevilacqua P, et al. Incremental yield of including Determine-TB LAM assay in diagnostic algorithms for hospitalized and ambulatory HIV-positive patients in Kenya. PLoS One **2017**; 12:e0170976.

26. Yee D, Valiquette C, Pelletier M, Parisien I, Rocher I, Menzies D. Incidence of serious side effects from first-line antituberculosis drugs among patients treated for active tuberculosis. Am J Respir Crit Care Med **2003**; 167:1472–1477.

27. Tweed CD, Crook AM, Amukoye EI, et al. Toxicity associated with tuberculosis chemotherapy in the REMoxTB study. BMC Infect Dis **2018**; 18:317.

28. Kranzer K, Lawn SD, Johnson LF, Bekker L-G, Wood R. Community viral load and CD4 count distribution among people living with HIV in a South African Township: implications for treatment as prevention. J Acquir Immune Defic Syndr **2013**; 63:498–505.

29. Barth RE, van der Loeff MFS, Schuurman R, Hoepelman AIM, Wensing AMJ. Virological follow-up of adult patients in antiretroviral treatment programmes in sub-Saharan Africa: a systematic review. Lancet Infect Dis **2010**; 10:155–166.

30. Brinkhof MWG, Pujades-Rodriguez M, Egger M. Mortality of patients lost to follow-up in antiretroviral treatment programmes in resource-limited settings: systematic review and meta-analysis. PLoS One **2009**; 4:e5790.

31. Bachani D, Garg R, Rewari BB, et al. Two-year treatment outcomes of patients enrolled in India’s national first-line antiretroviral therapy programme. Natl Med J India **2010**; 23:7–12.

32. Clinton Health Access Initiative. 2016 Antiretroviral (ARV) CHAI Reference Price List. Available at: https://clintonhealthaccess.org/wp-content/uploads/2016/11/2016-CHAI-ARV-Reference-Price-List_FINAL.pdf. Accessed 28 April 2020.

33. Clinton Health Access Initiative. 2017 Antiretroviral (ARV) CHAI Reference Price List. Available at: https://www.clintonhealthaccess.org/wp-content/uploads/2017/12/2017-CHAI-ARV-Reference-Price-List_FINAL.pdf. Accessed 8 October 2020.

34. Dugdale CM, Ciaranello AL, Bekker L-G, et al. Risks and benefits of dolutegravir- and efavirenz-based strategies for South African women with HIV of child-bearing potential: a modeling study. Ann Intern Med **2019**; 170:614–625.

35. The World Bank. PPP conversion factor, GDP. Available at: https://data.worldbank.org/indicator/PA.NUS.PPP. Accessed 28 April 2020.

36. Clinton Health Access Initiative. CHAI ARV Ceiling Price List: 2014. 2015. Available at: https://clintonhealthaccess.org/chai-arv-ceiling-price-list-2014/. Accessed 10 February 2020.

37. Claassens MM, du Toit E, Dunbar R, et al. Tuberculosis patients in primary care do not start treatment. What role do health system delays play? Int J Tuberc Lung Dis **2013**; 17:603–607.

38. Pepper DJ, Marais S, Bhaijee F, Wilkinson RJ, De Azevedo V, Meintjes G. Assessment at antiretroviral clinics during TB treatment reduces loss to follow-up among HIV-infected patients. PLoS One **2012**; 7:e37634.

39. Lawn SD, Kerkhoff A, Burton R, et al. Massive diagnostic yield of HIV-associated tuberculosis using rapid urine assays in South Africa [CROI abstract 811LB]. Top Antivir Med **2014**; 22:422.

40. Doubilet P, Begg CB, Weinstein MC, Braun P, McNeil BJ. Probabilistic sensitivity analysis using Monte Carlo simulation: a practical approach. Med Decis Making **1985**; 5:157–177.

41. Fenwick E, O’Brien BJ, Briggs A. Cost-effectiveness acceptability curves--facts, fallacies and frequently asked questions. Health Econ **2004**; 13:405–415.

42. Pei PP, Weinstein MC, Li XC, et al. Prioritizing HIV comparative effectiveness trials based on value of information: generic versus brand-name ART in the US. HIV Clin Trials **2015**; 16:207–218.

43. Statistics South Africa. Public healthcare: How much per person? Available at: http://www.statssa.gov.za/?p=10548. Accessed 28 April 2020.

44. Padarath A, Barron P, editors. South African Health Review 2017, 20th edition. 2017; Available at: http://www.hst.org.za/publications/Pages/HSTSouthAfricanHealthReview.aspx. Accessed 28 April 2020.

45. Shisana O, Hall E, Maluleke KR, et al. The Impact of HIV/AIDS on the Health Sector. National Survey of Health Personnel, Ambulatory and Hospitalised Patients and Health Facilities, 2002. 2003;

46. UNAIDS. Countries. Available at: http://www.unaids.org/en/regionscountries/countries. Accessed 28 April 2020.

47. Espinal MA, Kim SJ, Suarez PG, et al. Standard short-course chemotherapy for drug-resistant tuberculosis: treatment outcomes in 6 countries. JAMA **2000**; 283:2537–2545.

48. Nathanson E, Lambregts-van Weezenbeek C, Rich ML, et al. Multidrug-resistant tuberculosis management in resource-limited settings. Emerg Infect Dis **2006**; 12:1389–1397.

49. Isaakidis P, Varghese B, Mansoor H, et al. Adverse events among HIV/MDR-TB co-infected patients receiving antiretroviral and second line anti-TB treatment in Mumbai, India. PLoS One **2012**; 7:e40781.

50. Schnippel K, Firnhaber C, Berhanu R, Page-Shipp L, Sinanovic E. Direct costs of managing adverse drug reactions during rifampicin-resistant tuberculosis treatment in South Africa. Int J Tuberc Lung Dis **2018**; 22:393–398.

51. Brust JCM, Shah NS, van der Merwe TL, et al. Adverse events in an integrated home-based treatment program for MDR-TB and HIV in KwaZulu-Natal, South Africa. J Acquir Immune Defic Syndr **2013**; 62:436–440.

52. Shea KM, Kammerer JS, Winston CA, Navin TR, Horsburgh CR. Estimated rate of reactivation of latent tuberculosis infection in the United States, overall and by population subgroup. Am J Epidemiol **2014**; 179:216–225.

53. Marx FM, Dunbar R, Enarson DA, et al. The temporal dynamics of relapse and reinfection tuberculosis after successful treatment: a retrospective cohort study. Clin Infect Dis **2014**; 58:1676–1683.

54. World Health Organization. Choosing interventions that are cost effective (WHO-CHOICE). Available at: http://www.who.int/choice/en/. Accessed 28 April 2020.

55. National Health Laboratory Service. Available at: http://www.nhls.ac.za/. Accessed 28 April 2020.

56. Maheswaran H, Petrou S, Cohen D, et al. Economic costs and health-related quality of life outcomes of hospitalised patients with high HIV prevalence: a prospective hospital cohort study in Malawi. PLoS One **2018**; 13:e0192991.

57. Yazdanpanah Y, Losina E, Anglaret X, et al. Clinical impact and cost-effectiveness of co-trimoxazole prophylaxis in patients with HIV/AIDS in Côte d’Ivoire: a trial-based analysis. AIDS **2005**; 19:1299–1308.

**Supplementary Table 1. Additional model input parameters.**

| **Parameter** | **South Africa** | **Malawi** | **Reference** |
| --- | --- | --- | --- |
| TB treatment success rate^a^ |  |  |  |
| DS-TB | 95% | 95% | [47] |
| MDR-TB | 78% | 78% | [48] |
| Monthly probability of toxicity while on DS-TB treatment | 8.1% | 8.1% | [27] |
| Monthly probability of toxicity while on MDR-TB treatment | 11.3% | 11.3% | [49] |
| Cost of treating toxicity from TB drugs, USD | $180 | $170 | [50,51] |
| Monthly probability of reactivation of latent TB (range,  based on CD4 cell count) | 0.05%-0.7% | 0.05%-0.7% | [52] |
| Monthly probability of TB relapse (range, based on time  from treatment completion) | 0.33%-0.008% | 0.33%-0.008% | [53] |
| Monthly probability of LTFU from HIV care | 0.2%-1.6% | 0.2%-1.6% | [30,31] |
| Cost of second-line ART (PI-based regimen), monthly, USD | $31 | $31 | [36] |
| Cost of outpatient visit (TB or HIV care), USD | $2.50 | $0.50 | [54] |
| CD4 test cost, USD | $14 | $14 | [55,56] |
| HIV RNA test cost, USD | $26 | $26 | [55,56] |
| HIV care costs (non-ART, stratified by CD4 cell count),  monthly, USD | $17-$135 | $2-$15 | [11,57] |

Abbreviations: TB, tuberculosis; DS, drug-susceptible; MDR, multidrug-resistant; USD, 2017 US dollars; LTFU, loss to follow-up; ART, antiretroviral therapy; PI, protease inhibitor

^a^Treatment success reflects the proportion of treatment-adherent patients who attain cure (negative smear and culture at the end of treatment).

**Supplementary Table 2. Additional true-positive and false-positive tuberculosis results from adding urine lipoarabinomannan testing to sputum Xpert in unselected hospitalized patients with HIV.**

|  | **South Africa** | | **Malawi** | |
| --- | --- | --- | --- | --- |
|  | Additional true-positive TB results compared to *Xpert*, per 1,000 people tested | Additional false-positive TB results compared to *Xpert*, per 1,000 people tested | Additional true-positive TB results compared to *Xpert*, per 1,000 people tested | Additional false-positive TB results compared to *Xpert*, per 1,000 people tested |
| *Xpert+AlereLAM* | 70 | 9 | 49 | 11 |
| *Xpert+FujiLAM* | 96 | 18 | 70 | 22 |

Abbreviation: TB, tuberculosis. These model results are derived from our base case assumptions regarding country-specific prevalence of TB, probability of sputum provision, distribution of CD4 counts among patients, and sensitivity, specificity, and diagnostic yield of each testing strategy in those with CD4<200/µL and those with CD4≥200/µL.

| **Supplementary Table 3. One-way sensitivity analysis results.** | | |
| --- | --- | --- |
|  | **ICER**^a^ **(USD/YLS), South Africa** | **ICER**^a^ **(USD/YLS), Malawi** |
| **Base case** |  |  |
| *Xpert+AlereLAM* | dominated | dominated |
| *Xpert+FujiLAM* | 830 | 440 |
| **Sensitivity analyses** |  |  |
| **Underlying TB prevalence (base case: 29% [South Africa], 24% [Malawi])** | | |
| 15% |  |  |
| *Xpert+AlereLAM* | dominated | dominated |
| *Xpert+FujiLAM* | 870 | 460 |
| 20% |  |  |
| *Xpert+AlereLAM* | dominated | dominated |
| *Xpert+FujiLAM* | 850 | 450 |
| 25% |  |  |
| *Xpert+AlereLAM* | dominated | dominated |
| *Xpert+FujiLAM* | 840 | 440 |
| 30% |  |  |
| *Xpert+AlereLAM* | dominated | dominated |
| *Xpert+FujiLAM* | 830 | 440 |
| 35% |  |  |
| *Xpert+AlereLAM* | dominated | dominated |
| *Xpert+FujiLAM* | 830 | 440 |
| 40% |  |  |
| *Xpert+AlereLAM* | dominated | dominated |
| *Xpert+FujiLAM* | 830 | 440 |
|  |  |  |
| **Supplementary Table 3, continued.** |  |  |
|  | **ICER (USD/YLS), South Africa** | **ICER (USD/YLS), Malawi** |
| **Underlying MDR prevalence among those with TB (base case: 3% [South Africa], 1% [Malawi])** | | |
| South Africa: 1% / Malawi 0.5% |  |  |
| *Xpert+AlereLAM* | dominated | dominated |
| *Xpert+FujiLAM* | 830 | 440 |
| South Africa: 5% / Malawi 3% |  |  |
| *Xpert+AlereLAM* | dominated | dominated |
| *Xpert+FujiLAM* | 830 | 440 |
| South Africa: 7% / Malawi 5% |  |  |
| *Xpert+AlereLAM* | dominated | dominated |
| *Xpert+FujiLAM* | 830 | 440 |
| **Probability of empiric treatment (base case weighted average between *Xpert* and *Xpert+LAM*: 10%)**^b^ | | |
| 0% |  |  |
| *Xpert+AlereLAM* | dominated | dominated |
| *Xpert+FujiLAM* | 830 | 440 |
| 5% |  |  |
| *Xpert+AlereLAM* | dominated | dominated |
| *Xpert+FujiLAM* | 830 | 440 |
| 15% |  |  |
| *Xpert+AlereLAM* | dominated | dominated |
| *Xpert+FujiLAM* | 830 | 440 |
| 20% |  |  |
| *Xpert+AlereLAM* | dominated | dominated |
| *Xpert+FujiLAM* | 830 | 450 |

| **Supplementary Table 3, continued.** |  | |  |
| --- | --- | --- | --- |
|  | **ICER (USD/YLS), South Africa** | **ICER (USD/YLS), Malawi** | |
| **Probability of sputum provision (base case: 50%)** | | | |
| 30% |  |  | |
| *Xpert+AlereLAM* | dominated | dominated | |
| *Xpert+FujiLAM* | 830 | 440 | |
| 40% |  |  | |
| *Xpert+AlereLAM* | dominated | dominated | |
| *Xpert+FujiLAM* | 830 | 440 | |
| 60% |  |  | |
| *Xpert+AlereLAM* | dominated | dominated | |
| *Xpert+FujiLAM* | 830 | 440 | |
| 70% |  |  | |
| *Xpert+AlereLAM* | dominated | 440 | |
| *Xpert+FujiLAM* | 830 | 450 | |
| 80% |  |  | |
| *Xpert+AlereLAM* | dominated | 430 | |
| *Xpert+FujiLAM* | 830 | 460 | |
| 90% |  |  | |
| *Xpert+AlereLAM* | dominated | 420 | |
| *Xpert+FujiLAM* | 820^c^ | 480 | |
| **Probability of loss to follow-up from TB care (base case: 3.6% per month)** | | | |
| 0.5x base case (1.8%) |  |  | |
| *Xpert+AlereLAM* | dominated | dominated | |
| *Xpert+FujiLAM* | 820 | 440 | |
| 1.5x base case (5.4%) |  |  | |
| *Xpert+AlereLAM* | dominated | dominated | |
| *Xpert+FujiLAM* | 840 | 440 | |
| 2x base case (7.2%) |  |  | |
| *Xpert+AlereLAM* | dominated | dominated | |
| *Xpert+FujiLAM* | 850 | 450 | |

| **Supplementary Table 3, continued.** |  | |  |
| --- | --- | --- | --- |
|  | **ICER (USD/YLS), South Africa** | **ICER (USD/YLS), Malawi** | |
| **Mortality from untreated TB (base case: 8.6% per month)**^d^ |  |  | |
| 0.25x base case (2.15%) |  |  | |
| *Xpert+AlereLAM* | dominated | 440 | |
| *Xpert+FujiLAM* | 730 | 440 | |
| 0.5x base case (4.3%) |  |  | |
| *Xpert+AlereLAM* | dominated | dominated | |
| *Xpert+FujiLAM* | 780 | 440 | |
| 1.5x base case (12.9%) |  |  | |
| *Xpert+AlereLAM* | dominated | dominated | |
| *Xpert+FujiLAM* | 870 | 450 | |
| 2x base case (17.2%) |  |  | |
| *Xpert+AlereLAM* | dominated | dominated | |
| *Xpert+FujiLAM* | 880 | 450 | |
| **FujiLAM specificity (base case: CD4<200 = 94%, CD4≥200 = 98%)** | | | |
| 75% |  |  | |
| *Xpert+AlereLAM* | 860 | 450 | |
| *Xpert+FujiLAM* | 910 | 570 | |
| 80% |  |  | |
| *Xpert+AlereLAM* | 860 | 450 | |
| *Xpert+FujiLAM* | 880 | 540 | |
| 85% |  |  | |
| *Xpert+AlereLAM* | dominated | 450 | |
| *Xpert+FujiLAM* | 860 | 510 | |
| 90% |  |  | |
| *Xpert+AlereLAM* | dominated | 450 | |
| *Xpert+FujiLAM* | 850 | 480 | |

| **Supplementary Table 3, continued.** |  | |  |
| --- | --- | --- | --- |
|  | **ICER (USD/YLS), South Africa** | **ICER (USD/YLS), Malawi** | |
| **Cost of FujiLAM, USD (base case: USD6)** |  |  | |
| USD3 |  |  | |
| *Xpert+AlereLAM* | dominated | dominated | |
| *Xpert+FujiLAM* | 820 | 430 | |
| USD10 |  |  | |
| *Xpert+AlereLAM* | dominated | 450 | |
| *Xpert+FujiLAM* | 840 | 460 | |
| USD15 |  |  | |
| *Xpert+AlereLAM* | dominated | 450 | |
| *Xpert+FujiLAM* | 850 | 500 | |
| USD20 |  |  | |
| *Xpert+AlereLAM* | dominated | 450 | |
| *Xpert+FujiLAM* | 860 | 540 | |
| **Cost of ART, USD (base case: first line = USD11, second line = USD32)** | | | |
| 0.5x base case |  |  | |
| *Xpert+AlereLAM* | dominated | dominated | |
| *Xpert+FujiLAM* | 760 | 370 | |
| 0.75x base case |  |  | |
| *Xpert+AlereLAM* | dominated | dominated | |
| *Xpert+FujiLAM* | 790 | 410 | |
| **Cost of HIV care, USD, base case: $17-$135 in South Africa, $2-15 in Malawi** | | | |
| 0.5x base case |  |  | |
| *Xpert+AlereLAM* | dominated | 350 | |
| *Xpert+FujiLAM* | 550 | 350 | |
| 0.75x base case |  |  | |
| *Xpert+AlereLAM* | dominated | dominated | |
| *Xpert+FujiLAM* | 690 | 400 | |

|  | **ICER (USD/YLS), South Africa** | **ICER (USD/YLS), Malawi** |
| --- | --- | --- |
| **Xpert+LAM yield (base case: *Xpert+AlereLAM* CD4<200/µL = 62%, CD4≥200/µL = 35%; *Xpert+FujiLAM* CD4<200/µL = 70%, CD4≥200/µL = 47%)**^e^ | | |
| -20% |  |  |
| *Xpert+AlereLAM* | dominated | dominated |
| *Xpert+FujiLAM* | 950 | 500 |
| -10% |  |  |
| *Xpert+AlereLAM* | dominated | 460 |
| *Xpert+FujiLAM* | 870 | 460 |
| +10% |  |  |
| *Xpert+AlereLAM* | dominated | 430 |
| *Xpert+FujiLAM* | 810 | 440 |
| +20% |  |  |
| *Xpert+AlereLAM* | dominated | 430 |
| *Xpert+FujiLAM* | 810 | 430 |

**Supplementary Table 3, continued.**

Abbreviations: USD, 2017 US dollars; YLS, year-of-life saved; ICER, incremental cost-effectiveness ratio; TB, tuberculosis; MDR, multidrug-resistant.

^a^The ICER for each testing strategy is relative to the next least costly, non-dominated testing strategy, which is either *Xpert* or *Xpert+AlereLAM*.

^b^We assumed that empiric treatment probability would be higher with *Xpert* compared with Xpert+LAM strategies.

^c^In some cases in South Africa – for example, when sputum provision probability is high – the ICER trend is counterintuitive due to our calibration to STAMP trial results (see Model Calibration and Validation section of the Supplement).

^d^*Xpert* misses more cases of TB compared with Xpert+LAM strategies. When mortality from untreated TB is very high, those with untreated TB are more likely to die quickly and therefore not accrue TB and HIV healthcare costs. Therefore, the ICER of Xpert+LAM strategies is counterintuitively higher.

^e^The stated decreases and increases represent absolute percentage changes to the yields among CD4<200/µL and CD4≥200/µL groups.

**Supplementary Table 4. Clinical and economic outcomes and cost-effectiveness of alternative tuberculosis testing strategies, including parallel, solo, sequential, and CD4-stratified algorithms.**

| **South Africa** | | | | |
| --- | --- | --- | --- | --- |
| **Testing strategy**^a^ | **Life-years, discounted**^b^  **(undiscounted)** | **Lifetime costs, USD,**  **discounted**^b^**^,^**^c^ | | **ICER (USD/YLS)**^d^ |
| *AlereLAM alone* | 8.8 (13.1) | 8,200 | | -- |
| *AlereLAM alone; if positive, then Xpert*^e^ | 8.8 (13.1) | 8,210 | | DOMINATED^f^ |
| *Xpert alone* | 8.9 (13.2) | 8,230 | | 400 |
| *FujiLAM alone* | 9.2 (13.6) | 8,450 | | dominated^g^ |
| *FujiLAM alone; if positive then Xpert*^e^ | 9.2 (13.6) | 8,460 | | DOMINATED^f^ |
| *Xpert+AlereLAM* | 9.2 (13.7) | 8,500 | | dominated^g^ |
| *Xpert for CD4≥200 cells/µL, Xpert+AlereLAM for CD4<200 cells/µL* | 9.2 (13.7) | 8,510 | | dominated^g^ |
| *Xpert for CD4≥200 cells/µL, Xpert+FujiLAM for CD4<200 cells/µL* | 9.3 (13.8) | 8,590 | | dominated^g^ |
| *Xpert+FujiLAM* | 9.4 (13.9) | 8,640 | | **830** |
| **Malawi** | | | | |
| **Testing strategy**^a^ | **Life-years, discounted**^b^ **(undiscounted)** | **Lifetime costs, USD,**  **discounted**^b^**^,^**^c^ | | **ICER (USD/YLS)**^d^ |
| *AlereLAM alone* | 8.4 (12.6) | 3,490 | | -- |
| *AlereLAM alone; if positive, then Xpert*^e^ | 8.4 (12.6) | 3,500 | | DOMINATED^f^ |
| *Xpert alone* | 8.5 (12.7) | 3,540 | | dominated^g^ |
| *FujiLAM alone* | 8.7 (12.9) | 3,600 | | 420 |
| *FujiLAM alone; if positive then Xpert*^e^ | 8.7 (12.9) | 3,610 | | DOMINATED^f^ |
| *Xpert for CD4≥200 cells/µL, Xpert+AlereLAM for CD4<200 cells/µL* | 8.7 (13.0) | 3,630 | | dominated^g^ |
| *Xpert+AlereLAM* | 8.8 (13.1) | 3,640 | | dominated^g^ |
| *Xpert for CD4≥200 cells/µL, Xpert+FujiLAM for CD4<200 cells/µL* | 8.8 (13.1) | 3,660 | | dominated^g^ |
| *Xpert+FujiLAM* | 8.9 (13.3) | | 3,710 | **480** |

^a^Unless otherwise specified, the stated test(s) was performed on all patients, regardless of CD4 cell count.

^b^Discounted 3% per year [5].

^c^This reflects lifetime healthcare costs.

^d^The ICER is the difference between two strategies in discounted costs divided by the difference in discounted life-years. The displayed life-years and costs are rounded, but the ICER was calculated using non-rounded life-years and costs. We considered a strategy cost-effective if its ICER was less than USD940/YLS in South Africa and less than USD750/YLS in Malawi (the ICERs of second-line antiretroviral therapy in these countries).

^e^In these strategies, if LAM is positive, then Xpert is performed for rifampicin resistance testing.

^f^This indicates “strong dominance.” This strategy resulted in the same or fewer life-years and higher costs compared with another strategy.

^g^This indicates “weak dominance.” The ICER of *Xpert+AlereLAM* versus *Xpert* was higher (less attractive) than the ICER of *Xpert+FujiLAM* versus *Xpert+AlereLAM*, indicating an inefficient use of resources.

| **Supplementary Table 5. Clinical and economic outcomes and cost-effectiveness when Xpert Ultra is used instead of Xpert.** | | | | |
| --- | --- | --- | --- | --- |
| **South Africa** | | | | |
| **Testing strategy** | **Mortality at 2 years, %** | **Life years, discounted**^a^ **(undiscounted)** | **Cost, USD, discounted**^a^**^,^**^b^ | **ICER, USD/YLS**^c^ |
| *XpertUltra* | 35.0 | 9.0 (13.4) | 8,320 | - |
| *XpertUltra+AlereLAM* | 32.8 | 9.3 (13.8) | 8,560 | dominated^d^ |
| *XpertUltra+FujiLAM* | 31.7 | 9.4 (14.0) | 8,670 | 830 |
| **Malawi** | | | | |
| **Testing strategy** | **Mortality at 2 years, %** | **Life years, discounted**^a^ **(undiscounted)** | **Cost, USD, discounted**^a^**^,^**^b^ | **ICER, USD/YLS**^c^ |
| *XpertUltra* | 38.4 | 8.6 (12.8) | 3,580 | - |
| *XpertUltra+AlereLAM* | 36.7 | 8.8 (13.2) | 3,670 | 440 |
| *XpertUltra+FujiLAM* | 35.9 | 8.9 (13.3) | 3,720 | 450 |

Abbreviations: USD: 2017 US dollars; ICER: incremental cost-effectiveness ratio; YLS: year-of-life saved.

^a^Discounted 3% per year [5].

^b^This reflects lifetime healthcare costs.

^c^The ICER is the difference between two strategies in discounted costs divided by the difference in discounted life-years. The displayed life-years and costs are rounded, but the ICER was calculated using non-rounded life-years and costs. We considered a strategy cost-effective if its ICER was less than USD940/YLS in South Africa and less than USD750/YLS in Malawi (the ICERs of second-line antiretroviral therapy in these countries).

^d^This indicates “weak dominance.” The ICER of *XpertUltra+AlereLAM* versus *XpertUltra* was higher (less attractive) than the ICER of *XpertUltra+FujiLAM* versus *XpertUltra+AlereLAM*, indicating an inefficient use of resources.

**Supplementary Figure 1. Overview of tuberculosis states in the simulation model.**


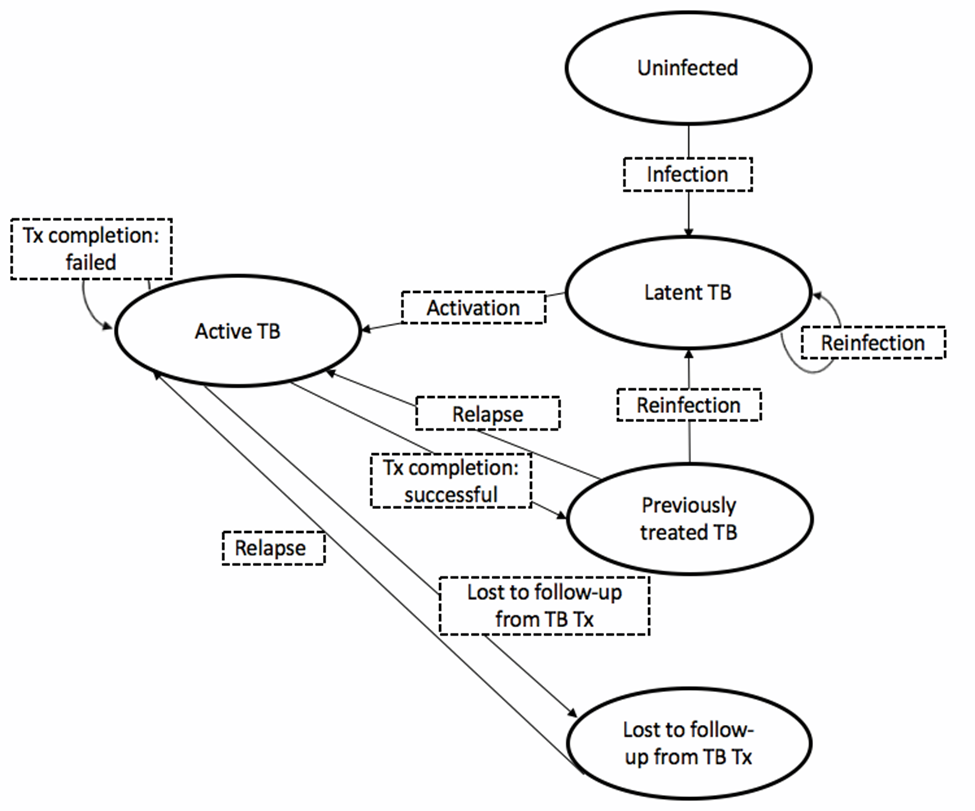


Abbreviation: Tx, treatment.

A simplified schematic of the tuberculosis natural history states and monthly transitions in the CEPAC-International model. Arrows that would indicate remaining in the current state are omitted for clarity. Individuals can transition from any of the displayed states to the Dead state (not shown). The figure excludes detail on tuberculosis drug resistance patterns and on HIV health states (CD4 cell count, antiretroviral therapy, and underlying tuberculosis risks). Some arrows reflect monthly transition probabilities, while others reflect overall probabilities. For example, a patient in the “Active TB” state has a one-time probability of treatment success, which can be attained upon completing a treatment course. However, while on treatment, the patient faces monthly probabilities of loss to follow-up and monthly probabilities of death.

**
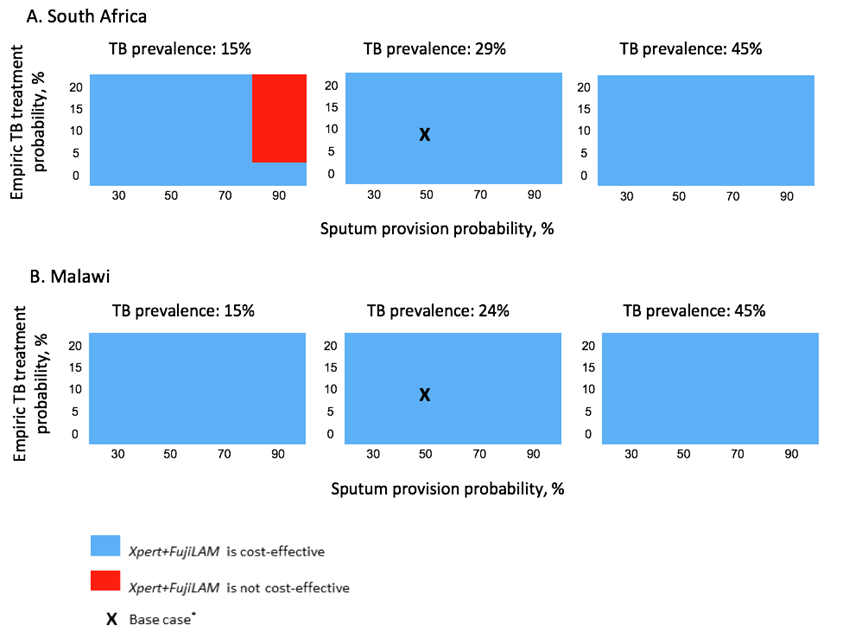
Supplementary Figure 2. Multi-way sensitivity analysis.**

Abbreviation: TB, tuberculosis.

These figures represent a three-way sensitivity analysis of tuberculosis prevalence (different heat maps), sputum provision probability (horizontal axis within each heat map), and empiric tuberculosis treatment probability (vertical axis within heat map). Results are shown for South Africa (A) and Malawi (B). The green areas are where *Xpert+FujiLAM* is cost-effective compared with *Xpert* and *Xpert+AlereLAM*, and it weakly dominates *Xpert+AlereLAM*. The red areas are where *Xpert+FujiLAM* is not cost-effective compared with *Xpert* or *Xpert+AlereLAM*.

^*^The base case values in each country are represented by the “X”. In the base case, South Africa and Malawi had different TB prevalence, but sputum provision probability and empiric tuberculosis treatment probability were the same in the two countries.

**
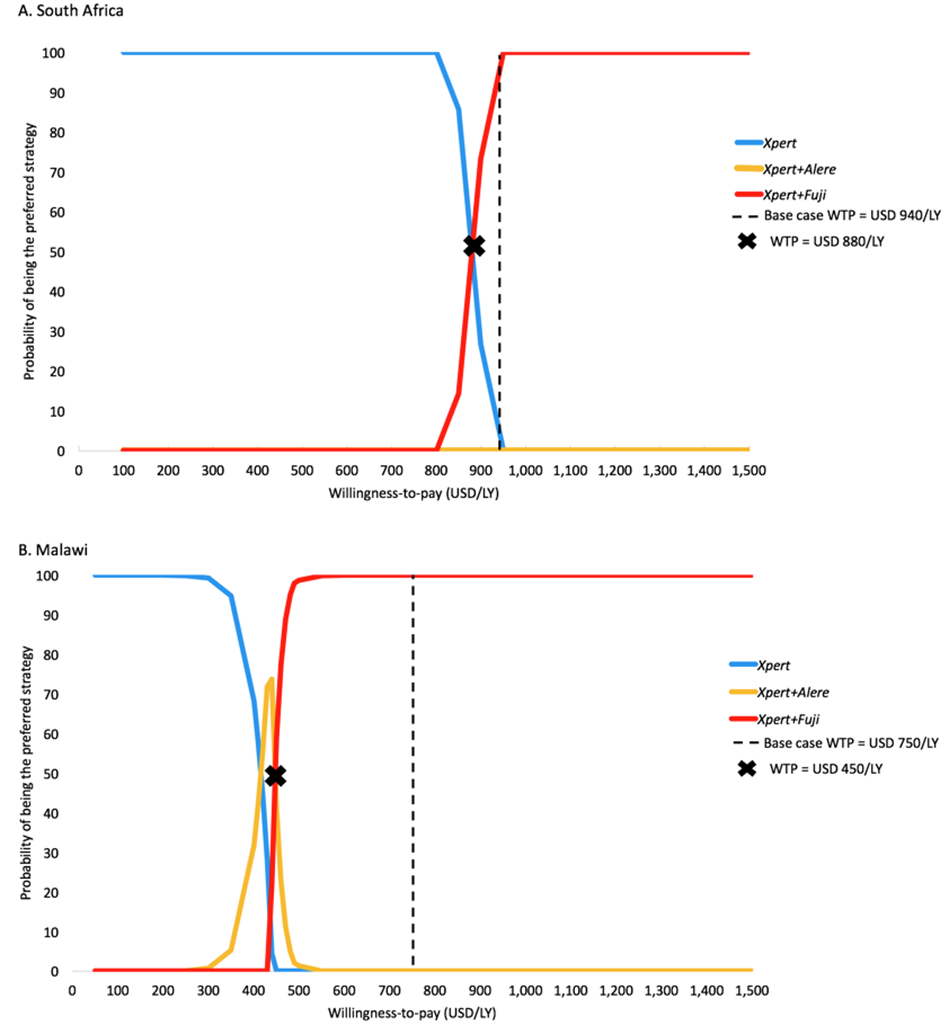
Supplementary Figure 3.** **Cost-effectiveness acceptability curves from probabilistic sensitivity analysis.**

Abbreviations: USD, 2017 US dollars; LY, life-year; WTP, willingness-to-pay.

These curves reflect results from a probabilistic sensitivity analysis in which we simultaneously varied tuberculosis prevalence, sputum provision, empiric tuberculosis treatment, loss to follow-up from tuberculosis care after hospitalization, and mortality from untreated tuberculosis. We determined the probability that a strategy would provide the highest net monetary benefit, defined as: (life-years)*(willingness-to-pay) – (lifetime costs). The vertical dashed line represents the base case willingness-to-pay (i.e., the cost-effectiveness threshold). The “X” marks the willingness-to-pay threshold beyond which *Xpert+FujiLAM* is the strategy most likely to provide the highest net monetary benefit.

**Supplementary**
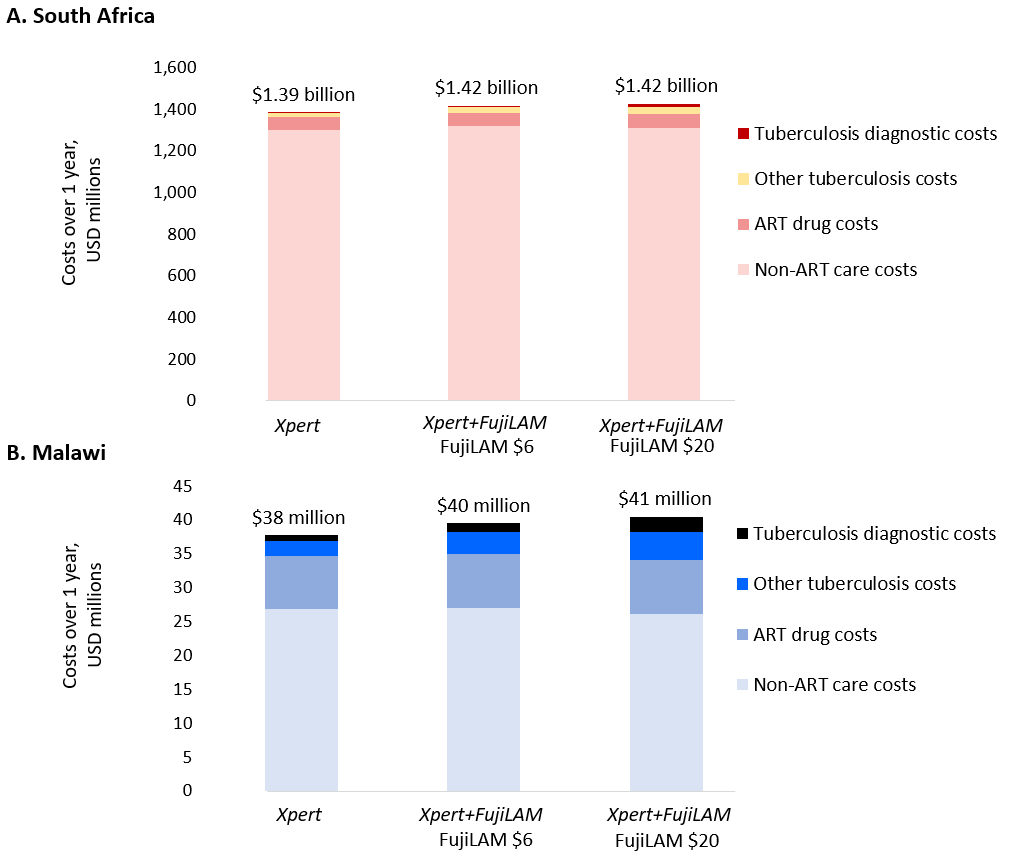
**Figure 4. Budget impact analysis at one-year horizon: implementing FujiLAM testing countrywide in South Africa and Malawi among hospitalized patients with HIV.**

Abbreviations: USD, 2017 US dollars; ART, antiretroviral therapy.

The vertical axis range is different between Panel A and Panel B. Budgetary projections are for the estimated 500,000 people with HIV who would be hospitalized in one year in South Africa and 70,000 people with HIV who would be hospitalized in one year in Malawi, all of whom would undergo tuberculosis testing. With each panel, the left bar represents one-year cumulative healthcare costs among these people if *Xpert* was the tuberculosis testing strategy. The middle bar reflects the *Xpert+FujiLAM* testing strategy, with FujiLAM costing USD6 per test. The right bar reflects the *Xpert+FujiLAM* testing strategy, with FujiLAM costing USD20 per test.
